# Fault Diagnosis in Analog Circuits Using Swarm Intelligence

**DOI:** 10.3390/biomimetics8050388

**Published:** 2023-08-25

**Authors:** Nadia Nedjah, Jalber Dinelli Luna Galindo, Luiza de Macedo Mourelle, Fernanda Duarte Vilela Reis de Oliveira

**Affiliations:** 1 Department of Electronics Engineering and Telecommunications, State University of Rio de Janeiro, Rio de Janeiro 20550-900, Brazil; jalber@marinha.mil.br; 2 Department of Systems Engineering and Computation, State University of Rio de Janeiro, Rio de Janeiro 20550-900, Brazil; ldmm@eng.uerj.br; 3 Department of Electronics and Computation, Polytechnic School, Federal University of Rio de Janeiro, Rio de Janeiro 21949-902, Brazil; fernanda.dvro@poli.ufrj.br

**Keywords:** analog circuits, fault diagnosis, swarm intelligence, Particle Swarm Optimization, Bat Algorithm

## Abstract

Open or short-circuit faults, as well as discrete parameter faults, are the most commonly used models in the simulation prior to testing methodology. However, since analog circuits exhibit continuous responses to input signals, faults in specific circuit elements may not fully capture all potential component faults. Consequently, diagnosing faults in analog circuits requires three key aspects: identifying faulty components, determining faulty element values, and considering circuit tolerance constraints. To tackle this problem, a methodology is proposed and implemented for fault diagnosis using swarm intelligence. The investigated optimization techniques are Particle Swarm Optimization (PSO) and the Bat Algorithm (BA). In this methodology, the nonlinear equations of the tested circuit are employed to calculate its parameters. The primary objective is to identify the specific circuit component that could potentially exhibit the fault by comparing the responses obtained from the actual circuit and the responses obtained through the optimization process. Two circuits are used as case studies to evaluate the performance of the proposed methodologies: the Tow–Thomas Biquad filter (case study 1) and the Butterworth filter (case study 2). The proposed methodologies are able to identify or at least reduce the number of possible faulty components. Four main performance metrics are extracted: accuracy, precision, sensitivity, and specificity. The BA technique demonstrates superior performance by utilizing the maximum combination of accessible nodes in the tested circuit, with an average accuracy of 95.5%, while PSO achieved only 93.9%. Additionally, the BA technique outperforms in terms of execution time, with an average time reduction of 7.95% reduction for the faultless circuit and an 8.12% reduction for the faulty cases. Compared to the machine-learning-based approach, using BA with the proposed methodology achieves similar accuracy rates but does not require any datasets nor any time-demanding training to proceed with circuit diagnostic.

## 1. Introduction

Devices that encompass electronic circuits are categorized into analog and digital circuits. According to statistics, almost 80% of electronic circuits in electronic devices are digital, but approximately 80% of failures primarily occur in the analog parts [1]. Digital circuits generally consist of components from a limited library of very simple models. When comparing digital circuits and analog circuits, fault diagnosis in digital circuits is easier than in analog circuits, as test procedures for digital circuits are well-defined, and only a limited number of faults, such as short-circuits, open circuits, and stuck-at faults, are present in digital circuits. In contrast, diagnosing faults in analog circuits is challenging due to the inherent characteristics of analog circuits, such as nonlinearity and component tolerances, inefficient fault models, inadequate accessible nodes, and measurement uncertainties. Therefore, more advancements are required for the diagnosis of faults in analog circuits.

A circuit failure is defined as an unauthorized deviation of at least one characteristic property or parameter of the system from the acceptable, usual, or standard condition [2]. In the field of analog circuits, failure is generally defined as a change in the nominal value of a parameter that significantly affects circuit performance, leading to circuit failure [3].

The main sources of failure in analog circuits are short circuits, open circuits, and component errors. Changes related to the connection of circuit component terminals result in both short circuit and open circuit failures [1]. A short circuit failure occurs when there is a short between the terminals of circuit components. An open circuit failure occurs when a component terminal does not make contact with other parts of the circuit. The disconnected terminal creates high resistance, leading to an open circuit failure. The components of an electronic circuit can also be the cause of circuit failures. If one of the circuit components is defective, the entire circuit becomes faulty due to the component failure. This component introduces an error in the circuit’s operation.

The development of testing strategies to detect and diagnose faults in analog and mixed-signal circuits is a challenging task that has spurred a significant amount of research due to the increasing number of applications for these circuits and the high cost of testing. Therefore, a strategy to detect and diagnose faults in these circuits is crucial [4].

In the past, an integrated circuit was merely a component in a system, but today the integrated circuit itself is the entire system. With this level of integration, this type of circuit has posed challenging problems for testing and design. Several factors contribute to the increased difficulties, including the lack of good fault models, a lack of a design standard with testability in mind, and the growing importance of timing-related faults [5]. As a result, the testing strategy for fault detection and diagnosis remains heavily reliant on the expertise and experience that engineers have regarding the circuit’s characteristics. Thus, fault detection and identification remain an interactive and time-consuming process [6]. Although there have been significant advancements, these new technologies have not been widely accepted.

The use of computational intelligence techniques for diagnosis is typically based on the construction of models or the use of classifiers. Of course, the success of model-based approaches depends on the quality of the yielded model, which can be challenging to obtain for complex systems. The computational intelligence-based approach, also known as data-driven fault diagnosis, exploits knowledge where fault diagnosis is performed based on available historical data with the aid of a classifier. Knowledge-based intelligence approaches are categorized as transformation-based, optimization-based, rule-based, machine learning-based, or hybrid techniques [1]:

- In the transformation-based approach, the model of the tested circuit is formulated, and the wavelet transform is applied to both fault-free and faulty circuit signals. A fault dictionary is constructed by extracting the standard deviation of the coefficients. The knowledge, along with the change in faulty parameters, is compared based on the fault dictionary to detect faults in the circuits [7].
- The optimization-based approach uses optimization algorithms to identify the component parameters. Nonlinear equations representing the tested circuit are considered as the optimization objective functions, and many optimization algorithms adapted for fault diagnosis techniques have been developed for fault detection in circuits. Fault detection is performed by comparing the estimated parameter with the reference values [8,9].
- In the rule-based approach, fault diagnosis in the circuit is performed based on rules in the form of “if symptoms, then fault.” Additional information is generated depending on the rules and signal domain information. Matching is performed based on this generated information [10,11].
- The machine learning-based approach exploits the knowledge from previous successful and/or unsuccessful diagnoses to improve the performance of the system in diagnostic procedures. In this case, the response of the circuit under test, with the component parameter value in the circuit without any excitation, is recorded and used as a pattern to train the exploited neural network. The variation of the parameter in the component, after the excitation, is tested using the neural network, which should allow revealing the faulty components of the circuit [1]. These kinds of models for circuit fault diagnostic are not perfect. Their faithfulness is measured by the delivered accuracy rates, achieved during the diagnostic process.
- Hybrid approaches are those that incorporate both machine learning-based and rule-based techniques for fault diagnosis in circuits [12].

In this work, the optimization-based approach is explored, where two swarm intelligence-inspired optimization techniques are used, allowing the detection of faults in analog electronic circuits based on impulse response. This is performed based on the analysis of the available circuit transfer functions. The experimentation part of this research work is conducted on second- and third-order electronic filters, namely Tow–Thomas Biquad [13] and Butterworth [14], respectively. These filters are flexible and can be used as low-pass, high-pass, and band-pass filters. For each circuit, case studies are performed to evaluate whether the optimization techniques classify the tested circuit either as faultless or faulty. In the latter, it identifies the possible faulty components in the tested circuit. In contrast with machine learning-based approaches, the proposed methodology does not need any prior datasets nor time-demanding training to proceed with analog circuit diagnostic. So, the proposed approach is more efficient in terms of computational time. Moreover, it achieves results with similar accuracy rates.

The remainder of this paper is organized into seven sections. Initially, in Section 2, we provide a literature review on fault diagnosis in analog electronic circuits, presenting techniques that utilize circuit analysis, classification, and optimization. Then, in Section 4, we present the two case studies that will be used in this investigation. Subsequently, in Section 3, we present some basic definitions of circuit analysis and the necessary tools to obtain the transfer functions of circuits. After that, in Section 5, we describe the transformation of fault diagnosis of an analog electronic circuit into an optimization problem and present case studies, introducing the methodologies used to detect fault-free circuits, circuits with a single fault, and circuits with multiple faults. In Section 6, we present the optimized swarming search techniques implemented in this work. Later, in Section 7, we define the testing methodology and the evaluation metrics and present and analyze the achieved results by the two investigated optimization techniques. We also offer a comparative evaluation regarding the performances of the techniques as well as with several classification-based approaches. Finally, in Section 8, we draw pertinent conclusions about which search strategy proved to be more suitable for analog circuit diagnosis. We also suggest some directions for future work improvement.

## 2. Related Works

This section presents recent works related to diagnosis and fault detection in analog electronic circuits. A fault is understood as a deviation in the value of a circuit component from its nominal value, which leads to a failure of the entire circuit [15]. Faults can be either catastrophic, as when the circuit exhibits open or short behavior, or parametric, as when degradation of a component occurs. Section 2.1 addresses recent works that used traditional circuit analysis techniques. Section 2.2 reviews the works that use classification as a tool for fault diagnosis. Section 2.3 presents related works that use optimization as a methodology for the detection and identification of faults in analog electronic circuits.

### 2.1. Circuit Analysis-Based Approach

In [16], a method is developed for fault localization and parameter identification in a linear circuit based on alternating current (AC) and direct current (DC). This method requires circuit analysis with nominal parameters and different excitations to measure the voltages at circuit nodes. To identify the fault, the mathematical technique known as the Woodbury Matrix Identity is used. The effectiveness of this methodology depends on the number of accessible nodes for measurement and the voltage differences between nodes in the nominal and perturbed circuits. In the latter case, it is possible for the voltage difference to be so small that it cannot be measured. In [17], a method is proposed for fault diagnosis in linear analog AC and DC circuits, as well as non-linear DC circuits with limited test points. The diagnosis includes soft fault testing of the circuits and identification of faulty elements. The method is based on the linear approximation of non-linear relationships; thus, it works if the parameter variations are small enough, requiring the analysis of two circuits that differ in their excitations. There is no guarantee that the faulty components will be identified in all cases. Sometimes, the set of potentially faulty elements obtained by the method is larger than the set of actually faulty elements.

In [18], an approach for fault diagnosis in analog circuits is presented, using MATLAB/Simulink modeling functionalities. The circuits are modeled in two scenarios: with and without faults. By using the signal flow graph, an input test stimulus is applied, and the presence of faults is identified by comparing the maximum measured output voltage to a predefined threshold. The choice of the voltage threshold for fault detection is one of the key parameters in this approach. The lower the voltage threshold, the higher the chance of detecting a fault. However, if the threshold is comparable to the noise voltage, a fault-free circuit may be misinterpreted as faulty. In [19], a method is developed for diagnosing mild faults in linear analog circuits, with a focus on circuits that feature current conveyors. This method exploits AC state measurement tests and utilizes nonlinear programming as a mathematical tool. The method has shown effectiveness in diagnosing single faults, but it exhibits lower efficiency in the case of multiple faults.

### 2.2. Classification-Based Approach

In traditional approaches, prior knowledge of the signal pattern model is required for fault diagnosis. However, in knowledge-based approaches, fault diagnosis is performed based on available historical data and with the assistance of classifiers [1]. In [20], a three-step approach technique is presented: testability calculation, fault localization and identification, and estimation of the faulty component value. The use of the MultiLayer Neural Network with Multi-Valued Neurons (MLMVN) as a classifier allows for a very precise association between the faulty component and its respective fault. In this work, a comparison of results is conducted between the classification techniques SVM and MLMVN, with MLMVN demonstrating better effectiveness. In [21], a combined method for fault diagnosis in analog circuits based on dependency matrices and intelligent classifiers is presented. The dependency matrix is calculated using sensitivity coefficients for accessible nodes. In this work, three types of classifiers are used for comparison: Backpropagation Neural Network, K-Nearest Neighbors (KNNs), and Random Forest (RF). The main objective of this method is to obtain groups of preliminary ambiguities through the construction of a dependency matrix, in order to achieve a more accurate diagnostic result using multiple simple intelligent classifiers. In addition, a new algorithm and optimization at the test point are proposed to simplify the input nodes, and the classifier is built for each separable ambiguity group.

In [22], a method for analog circuit fault diagnosis and unknown state recognition based on density peak clustering and a Voting Probabilistic Neural Network (VPNN) is proposed. Pattern recognition techniques such as KNN and density peak clustering procedures are also utilized to automatically determine the number of new neuron classes. In [23], an improved algorithm based on hierarchical Levenberg–Marquardt (LM) combined with Discrete Volterra Series (DVS) is proposed. The DVS is a common feature extraction method with challenging parameter estimation. In this algorithm, DVS is simplified based on the hierarchical symmetry of memory parameters. The LM strategy is used to optimize the coefficients. Additionally, a Bayesian information criterion based on entropy symmetry is introduced for order selection. For fault diagnosis, a method combining the improved DVS algorithm and condensed nearest neighbor algorithm is applied. The improved method showed to be useful for simplifying the calculation of DVS parameters for circuit faults in analog electronic systems. In [24], Decision Tree is used for fault diagnosis in analog electrical circuits. However, to increase the accuracy and efficiency of the Decision Tree, two improved trees are introduced: Cluster Validity-based Decision Tree (CV-DT) and Failure Rate-based Decision Tree (FR-DT). The CV-DT is constructed by an enhanced algorithm that considers the cluster validity index. This method selects the splitting attributes with higher classification credibility, thereby increasing the diagnosis accuracy. On the other hand, the FR-DT, constructed by an enhanced algorithm considering the failure rates, not only considers the partitioning capability of each attribute but also takes into account the prioritization of fault isolation with higher failure rates.

### 2.3. Optimization-Based Approach

The intelligence-based approach is also referred to as a data-driven fault diagnosis approach. It is categorized into transformation-based techniques, optimization-based techniques, machine learning techniques, hybrid techniques, and rule-based techniques [1]. In [25], a novel method for diagnosing parametric faults in analog circuits based on the circuit’s transfer function is presented. The Genetic Algorithm (GA) is used to optimize the system parameters. In this method, each system parameter is composed of multiple components, which are grouped into appropriate modules. The GA is then employed to design the filter using the system parameters and also diagnose the fault and locate it at the module level. In [26], a recursive method is presented for calculating higher-order sensitivities of node voltages in analog electronic circuits. Fault identification is achieved by solving the equations related to the deviations of the elements, which are obtained from the computation of sensitivity coefficients. To find the solution of these equations, the Particle Swarm Optimization (PSO) technique is exploited.

In [27], a simulation-based GA approach for fault diagnosis is proposed. Fault diagnosis is transformed into an optimization problem, where the genes represent the values of potential faulty component parameters. The faulty response of the circuit is the objective. The goal is to minimize the difference between the actual faulty response and the simulated response from the GA. This method does not save all possible faults in advance, although it can diagnose all continuous fault values. In [28], it is shown that the identification of parameters with faults is important for predicting the remaining useful life of the tested circuit. Based on the circuit’s transfer function, the measured faulty response is used to deduce the possible parameters with faults. It is known that the optimization response is a function of the analog parameters. Typically, the number of independent responses is much smaller than the number of analog components. Underdetermined equations have infinite solutions, meaning there are many combinations of analog parameters that can generate the same faulty response. The proposed method exploits an evolutionary process based on GA. The length of the chromosome is equal to the number of analog components, with each gene representing the parameter value of a component. Based on the gene values and the transfer function, each individual has a simulated response. The objective is to find all possible individuals that minimize the difference between the simulated and measured faulty responses.

## 3. Analog Circuit Analysis

In circuit analysis, it is possible to find voltages and currents in a circuit by applying a constant frequency input source. By keeping the amplitude of the source constant and varying the frequency, we obtain the frequency response of the circuit. The frequency response can be considered as a complete description of the steady-state behavior of a circuit as a function of frequency.

Ohm’s Law and Kirchhoff’s Laws are the fundamental laws of circuit theory. Reference [29] details how the application of these laws can be used to develop circuit analysis techniques. The techniques are nodal analysis and mesh analysis. With these techniques, any circuit can be analyzed by obtaining a set of simultaneous equations that are then solved to obtain the necessary values of current or voltage. As a result of this set of equations, the transfer function of the circuit in question is generated.

Nodal analysis is a tool used to analyze circuits using node voltages as circuit variables. Choosing node voltages is convenient and minimizes the number of equations that need to be solved simultaneously. Nodal analysis is also known as the node voltage method. Nodal analysis of a circuit with *n* nodes involves the following three steps:

- Select a reference node (ground node, 0 V). In the remaining nodes, assign variables $v_{1}$, $v_{2}$, …, $v_{n-1}$. The voltage equations will have the chosen reference node as the reference.
- Apply Kirchhoff’s laws at each of the n−1 non-reference nodes. Use Ohm’s law to express branch currents in terms of node voltages.
- Solve the resulting equations to obtain the node voltage with respect to the reference node.

A mesh is defined as a loop that does not contain any other internal loops. Mesh analysis is a tool that uses the currents of a mesh as circuit variables. Using mesh currents reduces the number of equations that need to be solved compared to using element currents. While nodal analysis utilizes Kirchhoff’s current law to find voltages, mesh analysis uses Kirchhoff’s voltage law to find the unknown currents. Mesh analysis has the limitation of being applicable only to circuits that do not have cross-branches, also known as planar circuits. Mesh analysis of a circuit with *n* meshes involves the following three steps:

- Assign mesh currents $i_{1}$, $i_{2}$, …, $i_{n}$ to the *n* meshes.
- Apply Kirchhoff’s voltage law to each of the *n* meshes. Use Ohm’s law to express voltages in terms of mesh currents.
- Solve the resulting equations to obtain the mesh currents.

The steady-state frequency response of circuits is important in many applications, especially in communication and control systems [29]. One specific application is in electrical filters that block or eliminate signals with unwanted frequencies and allow the passage of signals with desired frequencies.

The transfer function is defined as the ratio between the Laplace transform of the output and the input of a given system when the initial conditions are zero [30]. In the analysis of analog electronic circuits with single input and output, the transfer function can be found by relating the voltage of the output signal to the voltage of the input signal, as shown in Equation (1):

(1) $$H\left(s\right)=\frac{V_{out}\left(s\right)}{V_{in}\left(s\right)},$$

where $V_{out}\left(s\right)$ is the function that defines the output node being analyzed in the circuit and $V_{in}\left(s\right)$ is the input function, both in the Laplace domain.

Circuit analysis studies the behavior of current flow through an electrical circuit, allowing us to understand the influence of each electronic component on the circuit’s response to an input signal. The transfer function of a circuit is described with respect to the measured node being analyzed [28], as shown in Equation (2):

(2) $$H^{\left(T_{n}\right)}\left(s,x\right)=\frac{{\dot{U}}_{out}\left(s,x\right)}{{\dot{U}}_{in}},$$

where *n* refers to the number of accessible nodes in the circuit, $T_{n}$ represents the accessible node of the circuit, *x* represents the circuit parameters, and ${\dot{U}}_{out}$ is the function defining the output node being analyzed in the circuit and ${\dot{U}}_{in}$ is the input function, both in the Laplace domain. The transfer function from Equation (2) can also be represented by Equation (3):

(3) $$H^{\left(T_{n}\right)}\left(s,x\right)=\frac{a_{p}\left(x\right)s^{p}+a_{p-1}\left(x\right)s^{p-1}+\cdots +a_{0}\left(x\right)}{b_{q}\left(x\right)s^{q}+b_{q-1}\left(x\right)s^{q-1}+...+b_{0}\left(x\right)},$$

where $X=[x_{1}x_{2}\ldots x_{K}]$ corresponds to the admittance of the *K* components in the circuit and q≥p. In the frequency domain, where s=jw, it can be observed that the frequency response of the circuit is expressed in complex form, as shown in Equation (4):

(4) $$H^{\left(T_{n}\right)}\left(jw,x\right)=h_{Re}^{\left(T_{n}\right)}\left(jw,x\right)+jh_{Im}^{\left(T_{n}\right)}\left(jw,x\right),$$

where $h_{Re}^{\left(T_{n}\right)}\left(jw,x\right)$ and $h_{Im}^{\left(T_{n}\right)}\left(jw,x\right)$ represent the real and imaginary parts of $H^{\left(T_{n}\right)}\left(jw,x\right)$, respectively. With *n* as the number of nodes in the circuit being analyzed, the frequency response is given by the dataset, as shown in Equation (5):

(5) $$H\left(x\right)=[h_{Re}^{\left(1\right)}\left(x\right),h_{Im}^{\left(1\right)}\left(x\right),\ldots ,h_{Re}^{\left(T_{n}\right)}\left(x\right),h_{Im}^{\left(T_{n}\right)}\left(x\right)],$$

where the vector H(x) represents the transfer functions of each node in the circuit, with each node having a real and an imaginary component. Similarly, the measured response for the analyzed circuit is also expressed in real and imaginary components, as shown in Equation (6):

(6) $$U=[{\dot{u}}_{Re}^{\left(1\right)},{\dot{u}}_{Im}^{\left(1\right)},{\dot{u}}_{Re}^{\left(2\right)},{\dot{u}}_{Im}^{\left(2\right)},\ldots ,{\dot{u}}_{Re}^{\left(T_{n}\right)},{\dot{u}}_{Im}^{\left(T_{n}\right)}],$$

where **U** represents the data obtained from measurements in the circuit. The data H(x) and **U** have the same dimensions and are equally separable.

In circuit analysis using transfer function equations, it is common to analyze the impulse response of the circuit, as in the Laplace domain the impulse function used as the input has a unit value [30]. When ${\dot{U}}_{in}=1$, Equation (2) is defined as $H\left(s,x\right)={\dot{U}}_{out}\left(s,x\right)$. It is noteworthy to point out that, in this work, we use the transfer functions of the tested circuits to continue with its fault diagnosis.

## 4. Case Studies

In this section, the circuits used as case studies are presented, as well as the process of obtaining their respective transfer functions.

### 4.1. Case Study 1: Tow–Thomas Biquad Filter

**Figure 1:**
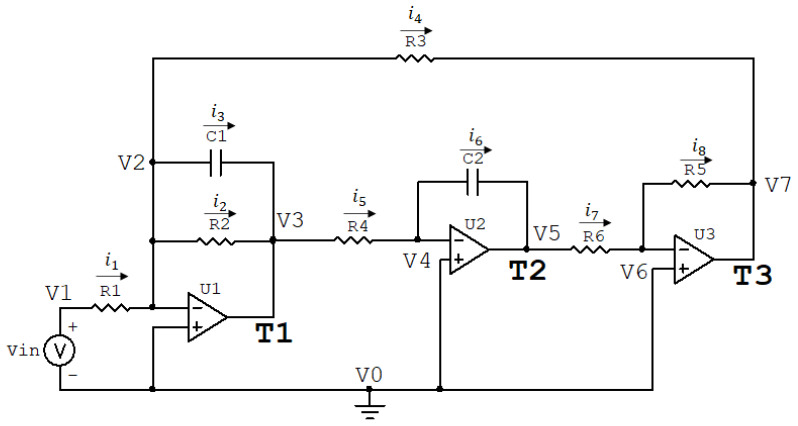
Schematics of the Tow–Thomas Biquad filter.

Figure 1 shows the circuit of the Tow–Thomas Biquad Filter. This circuit is a second-order active filter based on two-integrator topology [13].

The circuit parameters consist of the values assigned to the components. In this filter, depending on the parameters, the circuit can function as a low-pass, band-pass, high-pass, or all-pass filter, with different types of cutoff. Therefore, it offers high flexibility in its usage due to its ease of manipulation. The circuit is of the RC and Operational Amplifier (Op-Amp) type. According to Figure 1, the circuit has $X=[R_{1}$ $R_{2}$ $R_{3}$ $R_{4}$ $R_{5}$ $R_{6}$ $C_{1}$ $C_{2}]$ as components, $i_{1}$ to $i_{8}$ represent the currents in the components, $V_{in}$ is the input voltage, and $V_{0}$ to $V_{7}$ are the voltages at the circuit nodes. So, using the nodal and mesh analyses of the circuit, we can obtain the transfer function. By convention, the calculations will be made considering that the Op-Amps are ideal, meaning that there is no current at the input terminals and the voltages at the input terminals are equal for these components. Therefore, we have the following equations in the Laplace domain:

(7) $$\begin{array}{ccc} V_{0} & = & 0 \end{array}$$

(8) $$\begin{array}{ccc} V_{1}-V_{0} & = & V_{in}\Rightarrow V_{1}=V_{in} \end{array}$$

(9) $$\begin{array}{ccc} V_{2}-V_{1} & = & R_{1}*i_{1} \end{array}$$

(10) $$\begin{array}{ccc} V_{3}-V_{2} & = & R_{2}*i_{2} \end{array}$$

(11) $$\begin{array}{ccc} V_{3}-V_{2} & = & \frac{i_{3}}{sC_{1}} \end{array}$$

(12) $$\begin{array}{ccc} V_{4}-V_{3} & = & R_{4}*i_{5}, \end{array}$$

(13) $$\begin{array}{ccc} V_{5}-V_{4} & = & \frac{i_{6}}{sC_{2}} \end{array}$$

(14) $$\begin{array}{ccc} V_{6}-V_{5} & = & R_{6}*i_{7} \end{array}$$

(15) $$\begin{array}{ccc} V_{7}-V_{6} & = & R_{5}*i_{8} \end{array}$$

(16) $$\begin{array}{ccc} V_{7}-V_{2} & = & R_{3}*i_{4}. \end{array}$$

Due to the characteristics of the Op-Amps, it can be observed that $i_{5}=i_{6}=i_{7}=i_{8}$. Therefore, all of them can be replaced by $i_{5}$ in the calculation development. Based on Equations (13)–(16), we obtain Equation (17):

(17) $$V_{7}-V_{3}=\left(R_{4}+\frac{1}{sC_{2}}+R_{6}+R_{5}\right)*i_{5}.$$

At nodes $V_{2}$ and $V_{3}$ of the circuit in Figure 1, we have Equations (18) and (19), respectively:

(18) $$\begin{array}{ccc} i_{1} & = & i_{2}+i_{3}+i_{4} \end{array}$$

(19) $$\begin{array}{ccc} i_{5} & = & i_{2}+i_{3}. \end{array}$$

As explained in Section 3, obtaining the transfer function involves initially determining the selected input and output nodes of the circuit. For Figure 1, the generator $V_{in}$ is used as the input at node $V_{1}$. In this circuit, the output terminals of the Op-Amps were considered as accessible nodes. Therefore, there are three accessible nodes for data collection: node $T_{1}$ at $V_{3}$, node $T_{2}$ at $V_{5}$, and node $T_{3}$ at $V_{7}$. The transfer functions are obtained by manipulating the presented equations to find a relationship between $V_{3}$, $V_{5}$, and $V_{7}$ and $V_{in}$.

So, by using Equations (10)–(12) and (16)–(19), we obtain the transfer function of the circuit in Figure 1 with respect to node $T_{1}$ ($H^{\left(T_{1}\right)}\left(s\right)$), given by Equation (20):

(20) $$H^{\left(T_{1}\right)}\left(s\right)=\frac{V_{3}}{V_{in}}=\frac{\frac{s}{R_{1}*C_{1}}}{s^{2}+\left(\frac{1}{R_{2}*C_{1}}\right)*s+\left(\frac{R_{5}}{R_{3}*R_{4}*R_{6}*C_{1}*C_{2}}\right)}.$$

Using Equation (20), which provides the relationship between $V_{3}$ and $V_{in}$, and Equations (13) and (14), we can obtain the transfer function of the circuit in Figure 1 with respect to node $T_{2}$ ($H^{\left(T_{2}\right)}\left(s\right)$), given by Equation (21):

(21) $$H^{\left(T_{2}\right)}\left(s\right)=\frac{V_{5}}{V_{in}}=\frac{\frac{1}{R_{1}*R_{4}*C_{1}*C_{2}}}{s^{2}+\left(\frac{1}{R_{2}*C_{1}}\right)*s+\left(\frac{R_{5}}{R_{3}*R_{4}*R_{6}*C_{1}*C_{2}}\right)}.$$

Using Equation (21), which provides the relationship between $V_{5}$ and $V_{in}$, and Equations (15) and (16), we can obtain the transfer function of the circuit in Figure 1 with respect to node $T_{3}$ ($H^{\left(T_{3}\right)}\left(s\right)$), given by Equation (22):

(22) $$H^{\left(T_{3}\right)}\left(s\right)=\frac{V_{7}}{V_{in}}=\frac{\frac{-R_{5}}{R_{1}*R_{4}*R_{6}*C_{1}*C_{2}}}{s^{2}+\left(\frac{1}{R_{2}*C_{1}}\right)*s+\left(\frac{R_{5}}{R_{3}*R_{4}*R_{6}*C_{1}*C_{2}}\right)}.$$

### 4.2. Case Study 2: Butterworth Filter

**Figure 2:**
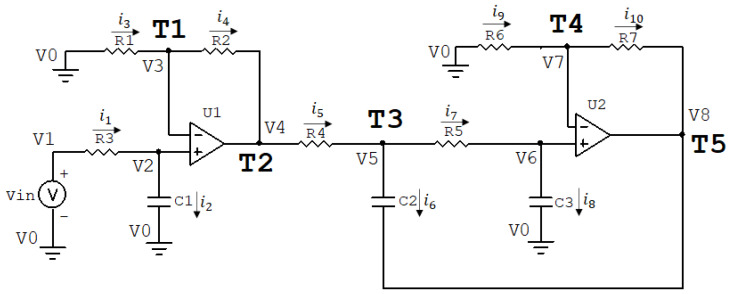
Schematics of the Butterworth filter.

Figure 2 shows the circuit of the Butterworth Filter. This circuit is a third-order active filter developed by [14]. Depending on the parameters, the circuit can function as a low-pass, band-pass, or high-pass filter, with different types of cutoff. Therefore, it offers high flexibility in its usage due to its ease of manipulation. This filter is widely used in biomedical applications, as signals need to be first amplified and filtered for further processing [31]. The circuit is of the RC and Operational Amplifier (Op-Amp) type.

According to Figure 2, the circuit has $X=[R_{1}$ $R_{2}$ $R_{3}$ $R_{4}$ $R_{5}$ $R_{6}$ $R_{7}$ $C_{1}$ $C_{2}$ $C_{3}]$ as components, $i_{1}$–$i_{10}$ represent the currents in the components, $V_{in}$ is the input voltage, and $V_{0}$ – $V_{8}$ are the node voltages. So, by performing nodal and mesh analyses of the circuit, we can obtain the transfer function. As a convention, the calculations will be made assuming ideal *Op-Amps*, meaning there is no current at the input terminals of these components. Based on Figure 2, we obtain the following equations in the Laplace domain:

(23) $$V_{0}=0,$$

(24) $$\begin{array}{ccc} V_{1}-V_{0} & = & V_{in}\;\;\;\;\;\;\;\;\Rightarrow V_{1}=V_{in} \end{array}$$

(25) $$\begin{array}{ccc} V_{2}-V_{0} & = & \frac{i_{2}}{sC_{1}}\;\;\;\;\;\;\Rightarrow V_{2}=\frac{i_{2}}{sC_{1}} \end{array}$$

(26) $$\begin{array}{ccc} V_{2}-V_{1} & = & R_{3}*i_{1} \end{array}$$

(27) $$\begin{array}{ccc} V_{3}-V_{0} & = & R_{1}*i_{3}\;\;\Rightarrow V_{3}=R_{1}*i_{3} \end{array}$$

(28) $$\begin{array}{ccc} V_{4}-V_{3} & = & R_{2}*i_{4} \end{array}$$

(29) $$\begin{array}{ccc} V_{5}-V_{4} & = & R_{4}*i_{5} \end{array}$$

(30) $$\begin{array}{ccc} V_{6}-V_{5} & = & R_{5}*i_{7} \end{array}$$

(31) $$\begin{array}{ccc} V_{6}-V_{0} & = & \frac{i_{8}}{sC_{3}}\;\;\;\;\;\;\Rightarrow V_{6}=\frac{i_{8}}{sC_{3}} \end{array}$$

(32) $$\begin{array}{ccc} V_{7}-V_{0} & = & R_{6}*i_{9}\;\;\Rightarrow V_{7}=R_{6}*i_{9} \end{array}$$

(33) $$\begin{array}{ccc} V_{8}-V_{5} & = & \frac{i_{6}}{sC_{2}} \end{array}$$

(34) $$\begin{array}{ccc} V_{8}-V_{7} & = & R_{7}*i_{10}. \end{array}$$

Based on the characteristics of the operational amplifiers, we can conclude that current $i_{1}=i_{2}$, current $i_{3}=i_{4}$, current $i_{7}=i_{8}$, and current $i_{9}=i_{10}$. All of these can be replaced by $i_{1}$, $i_{3}$, $i_{7}$, and $i_{9}$, respectively, in the following development. Based on Equations (28)–(30), we have $V_{5}=\left(R_{1}+R_{2}+R_{4}\right)*i_{3}$. Furthermore, at node $V_{5}$ of the circuit in Figure 2, we have $i_{5}=i_{6}+i_{7}$.

As explained in Section 3, the transfer function is obtained by initially determining the nodes considered as input and output of the circuit. For the circuit in Figure 2, the generator $V_{in}$ is used as the input at node $V_{1}$. In this circuit, there are five accessible nodes for data collection: node $T_{1}$ with $V_{3}$, node $T_{2}$ with $V_{4}$, node $T_{3}$ with $V_{5}$, node $T_{4}$ with $V_{7}$, and node $T_{5}$ with $V_{8}$. The transfer functions are obtained by manipulating the presented equations to find a relationship between $V_{3}$, $V_{4}$, $V_{5}$, $V_{7}$, $V_{8}$, and $V_{in}$.

Using Equations (25)–(27) and (31)–(34), and taking into account the aforementioned current equations, the transfer function of the circuit in Figure 2 with respect to node $T_{1}$ ($H^{\left(T_{1}\right)}\left(s\right)$) is obtained, given by Equation (35):

(35) $$H^{\left(T_{1}\right)}\left(s\right)=\frac{V_{3}\left(s\right)}{V_{in}}=\frac{B_{\left(T_{1},2\right)}*s^{2}+B_{\left(T_{1},1\right)}*s+B_{\left(T_{1},0\right)}}{s^{3}+B_{2}*s^{2}+B_{1}*s+B_{0}},$$

where the factors $B_{\left(T_{1},0\right)}$ to $B_{\left(T_{1},2\right)}$ are defined in Equations (37) and (38):

(36) $$\begin{array}{ccc} B_{\left(T_{1},0\right)} & = & \frac{1}{R_{1}*R_{4}*R_{5}*C_{1}*C_{2}*C_{3}} \end{array}$$

(37) $$\begin{array}{ccc} B_{\left(T_{1},1\right)} & = & -\frac{R_{4}*R_{7}*C_{2}+\left(R_{4}+R_{5}\right)*R_{6}*C_{3}}{R_{1}*R_{5}*R_{6}*C_{1}*C_{3}} \end{array}$$

(38) $$\begin{array}{ccc} B_{\left(T_{1},2\right)} & = & \frac{1}{R_{1}*C_{1}}. \end{array}$$

Using Equation (35), which provides the relationship between $V_{3}$ and $V_{in}$, and Equations (28) and (29), we obtain the transfer function of the circuit in Figure 2 with respect to node $T_{2}$ ($H^{T_{2}}\left(s\right)$), given by Equation (39):

(39) $$H^{T_{2}}\left(s\right)=\frac{V_{4}\left(s\right)}{V_{in}}=\frac{B_{\left(T_{2},2\right)}*s^{2}+B_{\left(T_{2},1\right)}*s+B_{\left(T_{2},0\right)}}{s^{3}+B_{2}*s^{2}+B_{1}*s+B_{0}},$$

where the factors $B_{\left(T_{2},0\right)}$ to $B_{\left(T_{2},2\right)}$ are defined in Equations (41) and (42).

(40) $$\begin{array}{ccc} B_{\left(T_{2},0\right)} & = & \frac{\left(R_{2}+R_{3}\right)}{R_{1}*R_{2}*R_{4}*R_{5}*C_{1}*C_{2}*C_{3}} \end{array}$$

(41) $$\begin{array}{ccc} B_{\left(T_{2},1\right)} & = & \frac{1}{R_{1}*R_{2}*C_{1}}\left(-\frac{\left(R_{2}+R_{3}\right)*R_{7}}{R_{5}*R_{6}*C_{3}}+\frac{R_{2}+R_{3}*R_{5}}{R_{4}*C_{2}}+\frac{R_{2}+R_{3}}{R_{5}*C_{2}}\right) \end{array}$$

(42) $$\begin{array}{ccc} B_{\left(T_{2},2\right)} & = & \frac{1}{R_{1}*C_{1}}\left(1+\frac{R_{3}}{R_{2}}\right). \end{array}$$

Using Equation (39), which provides the relationship between $V_{4}$ and $V_{in}$, and Equations (29) and (30), we obtain the transfer function of the circuit in Figure 2 with respect to node $T_{3}$ ($H^{\left(T_{3}\right)}\left(s\right)$), given by Equation (43):

(43) $$H^{\left(T_{3}\right)}\left(s\right)=\frac{V_{5}\left(s\right)}{V_{in}}=\frac{\left(\frac{\left(R_{3}+R_{2}\right)\left(1+R_{5}*C_{3}*s\right)}{R_{1}*R_{2}*R_{4}*R_{5}*C_{1}*C_{2}*C_{3}}\right)}{s^{3}+B_{2}*s^{2}+B_{1}*s+B_{0}},$$

Using Equation (43), which provides the relationship between $V_{5}$ and $V_{in}$, and Equations (31) and (32), and the voltage $V_{5}=\left(R_{1}+R_{2}+R_{4}\right)*i_{3}$, we obtain the transfer function of the circuit in Figure 2 with respect to node $T_{4}$ ($H^{\left(T_{4}\right)}\left(s\right)$), given by Equation (44):

(44) $$H^{\left(T_{4}\right)}\left(s\right)=\frac{V_{7}\left(s\right)}{V_{in}}=\frac{\frac{R_{3}+R_{2}}{R_{1}*R_{2}*R_{4}*R_{5}*C_{1}*C_{2}*C_{3}}}{s^{3}+B_{2}*s^{2}+B_{1}*s+B_{0}},$$

Using Equation (44), which provides the relationship between $V_{7}$ and $V_{in}$, and Equations (33) and (34), we obtain the transfer function of the circuit in Figure 2 with respect to node $T_{5}$ ($H^{\left(T_{5}\right)}\left(s\right)$), given by Equation (45):

(45) $$H^{\left(T_{5}\right)}\left(s\right)=\frac{V_{8}\left(s\right)}{V_{in}}=\frac{\frac{\left(R_{2}+R_{3}\right)*\left(R_{6}+R_{7}\right)}{R_{1}*R_{2}*R_{4}*R_{5}*R_{6}*C_{1}*C_{2}*C_{3}}}{s^{3}+B_{2}*s^{2}+B_{1}*s+B_{0}},$$

In Equations (35), (39), (43), (44), and (45), the factors $B_{0}$ to $B_{2}$ are defined in Equations (47) and (48):

(46) $$\begin{array}{ccc} B_{0} & = & \frac{1}{C_{3}*C_{2}*C_{1}*R_{1}*R_{4}*R_{5}} \end{array}$$

(47) $$\begin{array}{ccc} B_{1} & = & \frac{1}{R_{4}*R_{5}*C_{2}*C_{3}}-\frac{R_{7}}{R_{1}*R_{5}*R_{6}*C_{1}*C_{3}}+\frac{R_{4}+R_{5}}{R_{1}*R_{4}*R_{5}*C_{1}*C_{2}} \end{array}$$

(48) $$\begin{array}{ccc} B_{2} & = & -\frac{R_{7}}{C_{3}*R_{5}*R_{6}}+\frac{R_{4}+R_{5}}{R_{4}*R_{5}*C_{2}}+\frac{1}{C_{1}*R_{1}}. \end{array}$$

## 5. Proposed Optimization Model for Fault Circuit Diagnostics

Given an analog electronic circuit with *K* components and *n* accessible nodes, termed $T_{i}$, for i=1…n, the transfer function can be defined as the voltage at an accessible node of the circuit when a unit impulse function is applied in the Laplace domain. When a sinusoidal voltage is applied from a signal generator with unit amplitude, the Laplace transform guarantees that the transfer function has a value equal to the voltage at the desired node, as stated in [30]. In the frequency domain, where s=jw, it can be observed that the circuit’s frequency response is expressed in complex form, as shown in Equation (4).

The measured response in the circuit at the *n* accessible points is also expressed in terms of real and imaginary components, as shown in Equation (6). The data H(x) and **U** have the same dimension and are equally separable. With the real and imaginary terms of H(x) and **U**, we have a system of 2n equations, as shown in Equation (49):

(49) $$\left\{\begin{array}{ccc} h_{Re}^{\left(T_{1}\right)}\left(x\right) & = & {\dot{u}}_{Re}^{\left(T_{1}\right)}\\ h_{Im}^{\left(T_{1}\right)}\left(x\right) & = & {\dot{u}}_{Im}^{\left(T_{1}\right)}\\ \cdots & \; & \\ h_{Re}^{\left(T_{n}\right)}\left(x\right) & = & {\dot{u}}_{Re}^{\left(T_{n}\right)}\\ h_{Im}^{\left(T_{n}\right)}\left(x\right) & = & {\dot{u}}_{Im}^{\left(T_{n}\right)} \end{array}\right.$$

The objective of the optimization is to find values of the components in **X** that satisfy Equation (49) such that the absolute difference between the measured voltage in the circuit and the voltage obtained by calculating the transfer function is minimized, as shown in Equation (50):

(50) $$\min_{X}E=||H\left(X\right)-U||,$$

where **X** represents the values of the circuit components that have the potential to exhibit faults, H(X) is the objective term that is calculated using the transfer function, and **U** is the term obtained by measuring the circuit under analysis.

The optimization results are characterized by $X^{*}$ as the best values of the circuit components that minimize the objective function. Fault diagnosis is performed by comparing the component values in $X^{*}$ with the specified operating range provided by the component manufacturer. The normal operating range used in the experiments presented in the following sections of this chapter was ±5% of the nominal value, as shown in Equation (51):

(51) $$f_{x_{K}}=\left\{\begin{array}{cc} 0, & 0.95*x_{K,ideal}\leq x_{K}^{*}\leq 1.05*x_{K,ideal}\\ \; & \\ 1, & x_{K}^{*}<0.95*x_{K,ideal}oux_{K}^{*}>1.05*x_{K,ideal}, \end{array}\right.$$

where $f_{x_{K}}$ identifies whether component *K* has a fault ($f_{x_{K}}=1$) or is non-defective ($f_{x_{K}}=0$), and $x_{K}$ refers to the ideal value of the analyzed component. It is noteworthy to emphasize that the optimization process used to discover faulty components uses no circuit simulation. Only the circuit transfer functions, which can be obtained automatically by abundantly available tools, are required to compose the objective function.

In the remainder of this section, three experiments regarding the two case studies are presented: an experiment of a circuit without faults, where the components have values considered ideal for operation, an experiment of a circuit with one faulty component while the remaining components have simulated values considered ideal, and an experiment with a circuit exhibiting faults in two components. For these case studies, the frequency assigned to the voltage source at the input of the circuit was 1kHz, with an amplitude of 1 V. For the first case study, the ideal component values $X_{\mathrm{ideal}}$ are defined as 10kΩ for the resistors and 10nF for the capacitors.

### 5.1. Application to Case Study 1

**Figure 3:**
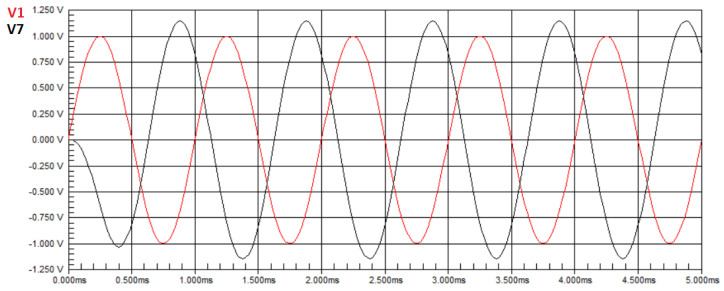
Waveform for the faultless Tow–Thomas Biquad filter.

Figure 3 shows the waveform of the circuit in Figure 1 without faults. The red sine wave represents the input signal applied by a signal generator, and the black sine wave represents the signal obtained at node $V_{7}$, also referred to as node $T_{3}$, which is assigned as the output in this case study.

It is observed that there is an amplification of the output signal voltage compared to the input signal, and the maximum voltage value obtained from the measurement in the circuit was $V_{7}=1.1460$ V. In Equation (50), U=1.1460. The transfer function at node $T_{3}$ of the Tow–Thomas Biquad circuit, in the Laplace domain, is given by Equation (22). Applying the ideal component values to Equation (22), we obtain $H^{T_{3}}\left(jw\right)=1.1463$. In Equation (50), we have H(X)=1.1463. With the values of H(X) and **U** obtained, the result of the minimization is E=0.0003. Hence, we conclude that the obtained values are equal to the defined nominal values.

**Figure 4:**
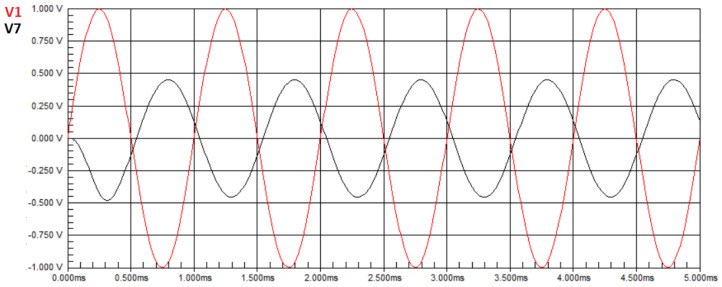
Waveform for the Biquad Tow–Thomas filter with faulty $R_{3}$.

For this second experiment, we simulate a single fault. The component values assigned are the ones considered ideal: 10kΩ for the resistors and 10nF for the capacitors, as before, except for one component, namely $R_{3}$, that has its value altered to a value outside the operating range, namely 4kΩ. Figure 4 presents the waveform of the circuit. It is observed that there is a loss of amplitude in the output signal voltage compared to the input signal, and the maximum voltage value obtained from the measurement in the circuit was $V_{7}=454.93$ mV. In Equation (50), we have U=0.45493. By applying the ideal component values to the transfer function in Equation (22), we obtain $H^{\left(T_{3}\right)}\left(jw\right)=1.1463$. In Equation (50), we have H(X)=1.1463. Therefore, the objective function of Equation (50) has a value of E=0.69107. Since the goal of the optimization is to search for a value close to zero, this value is inappropriate and the algorithm will search for new solutions, resulting in a lower objective function value. However, when applying values of the components as defined for this second experiment to the transfer function in Equation (22), we obtain $H^{\left(T_{3}\right)}\left(jw\right)=0.45517$. In Equation (50), we have H(X)=0.45517. For these values, the objective function has the near-zero value of E=0.00024.

**Figure 5:**
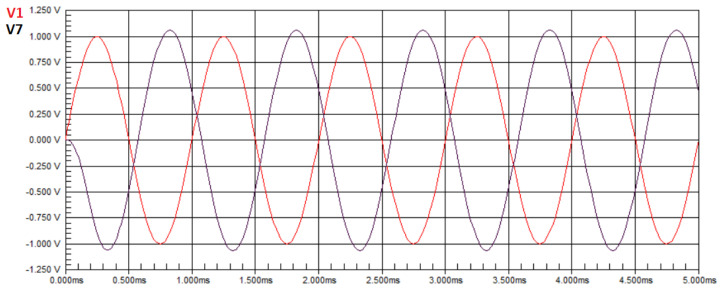
Waveform for the Biquad Tow–Thomas filter with faulty $R_{2}$ and $C_{2}$.

For this third experiment, we simulate a case of multiple faults. The component values assigned are the ones considered ideal: 10kΩ for the resistors and 10nF for the capacitors, except for two components, namely $R_{2}$ and $C_{2}$, that have their values altered to values outside the operating range, namely 6kΩ and 4nF, respectively. Figure 5 presents the waveform of the circuit with these two faults.

It can be observed that there is a gain in amplitude in the output signal voltage compared to the input signal, and the voltage value obtained from the measurement in the circuit was $V_{7}=1.0634$ V. In Equation (50), U=1.0634. By applying the ideal values to the transfer function in Equation (22), we obtain $H^{\left(T_{3}\right)}\left(jw\right)=1.1463$. In Equation (50), we have H(X)=1.1463. For these values, the objective function in Equation (50) provides a value of E=0.0829. Since the goal of the optimization is to search for a value close to zero, this value is inappropriate, and the algorithm will search for new solutions, resulting in a lower objective function value. However, when applying component values for resistors defined for this case to the transfer function in Equation (22), we obtain $H^{\left(T_{3}\right)}\left(jw\right)=1.0632$. In Equation (50), we have H(X)=1.0632. For these values, the objective function in Equation (50) has the near-zero value of E=0.0002.

### 5.2. Application to Case Study 2

**Figure 6:**
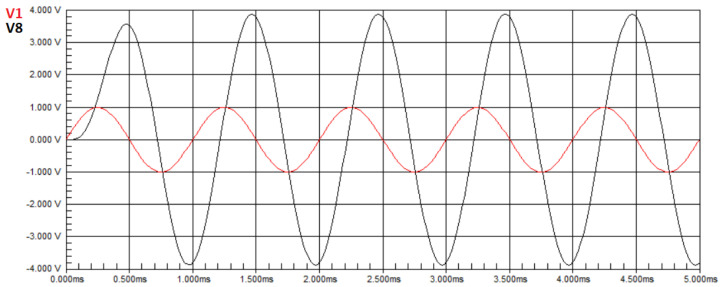
Waveform for the faultless Butterworth filter.

Figure 6 presents the waveform of the circuit from Figure 2 without any faults, where the red sine wave represents the input signal applied by a signal generator, and the black sine wave represents the signal obtained at node $V_{8}$, also referred to as node $T_{5}$, assigned as the output in this case study.

We can observe that there is an amplification in the voltage amplitude of the output signal compared to the input signal, and the measured voltage in the circuit is $V_{8}=3.8810$ V. In other words, in Equation (50), U=3.8810. The transfer function at node $T_{5}$ of the Butterworth circuit, in the Laplace domain, is given by Equation (45). Applying the component values of the circuit in Equation (45), with a frequency of 1 kHz, we obtain $H^{\left(T_{5}\right)}\left(jw\right)=3.8823$. Therefore, according to Equation (50), H(X)=3.8823. By substituting the values of H(X) and **U** into Equation (50), the result of the minimization is E=0.0013. Hence, we confirm that the obtained values are equal to the defined nominal values.

**Figure 7:**
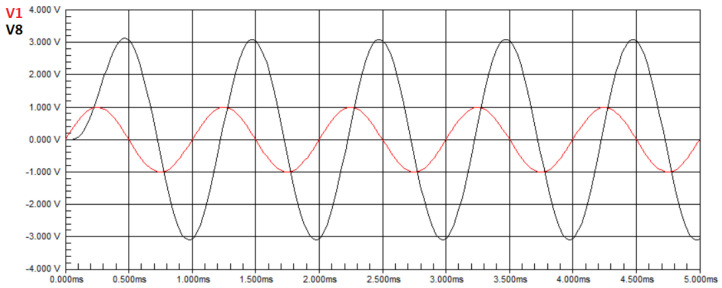
Waveform for the Butterworth filter with faulty $C_{2}$.

For this second experiment, wherein we simulate the case of a single fault, the component values assigned are the ideal values, except for one component, namely $C_{2}$, that has its value changed to be outside the operating range, namely 7nF. Figure 7 presents the waveform of the circuit with a fault.

It is observed that there is an amplitude gain in the output voltage compared to the input signal, although it is lower than the one observed in the fault-free experiment of this circuit. The measured voltage in the circuit is $V_{7}=3.0973$ V. In Equation (50), we have U=3.0973. By applying the component ideal values in the transfer function of Equation (45), we obtain $H^{\left(T_{5}\right)}\left(jw\right)=3.8823$. In Equation (50), we also have H(X)=3.8823. For these values, the objective function in Equation (50) provides E=0.785. Since the optimization goal is to find a value close to zero, this value is inappropriate, and the algorithm will search for new solutions that result in a lower objective function value. However, when the component values are set up as for this second experiment, in the transfer function of Equation (45), we obtain $H^{\left(T_{5}\right)}\left(jw\right)=3.1037$. In Equation (50), we compute H(X)=3.1037. For these values, the objective function has the near-zero value of E=0.0064.

**Figure 8:**
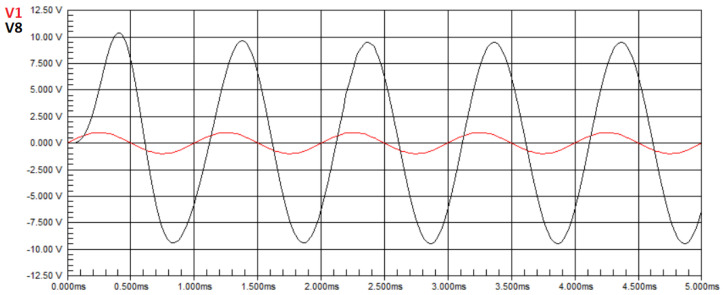
Waveform for the Butterworth filter with faulty $R_{1}$ and $C_{3}$.

For this third experiment, wherein we simulate the case of multiple faults, the values assigned to the components are the ideal values, except for two components, namely $R_{1}$ and $C_{3}$, that have their values changed outside the operating range, namely 3kΩ and 6nF, respectively. Figure 8 presents the waveform of the circuit with these faults.

It can be observed that there is a gain in amplitude in the output voltage compared to the input signal, albeit lower than the one observed in the fault-free experiment of this circuit, and the voltage value obtained by measuring the circuit was $V_{7}=9.4793$ V. In Equation (50), we have U=9.4793. As before, for the faultless circuit, we obtain $H^{\left(T_{5}\right)}\left(jw\right)=H\left(X\right)=3.8823$. For these values, the objective function in Equation (50) provides E=5.597. Since the optimization objective is to find a value close to zero, this value is not suitable, and the algorithm will search for new solutions that result in a lower value of the objective function. However, when the component values are set up as for this third experiment, in the transfer function of Equation (45), we obtain $H^{\left(T_{5}\right)}\left(jw\right)=9.4892$. In Equation (50), we compute H(X)=9.4883. For these values, the objective function is the near-zero value of E=0.0009.

## 6. Swarm Intelligence-Based Search Strategies

Swarm intelligence techniques offer several advantages when applied to optimization problems. They combine global exploration by searching a wide solution space, with local exploitation by refining solutions around promising areas. This balance helps them avoid getting stuck in local optima and increases the likelihood of finding better solutions. Moreover, individual agents in swarm intelligence algorithms typically follow simple heuristics. This simplicity often translates to efficient computation and easy implementation, making these techniques applicable to a wide range of optimization problems. These techniques are applicable to optimization problems where derivatives are hard to compute. Furthermore, they can handle non-convex and complex solution spaces, where traditional optimization methods might struggle due to the presence of multiple local optima and complex interactions between variables. Swarm intelligence techniques can be scaled up to tackle large-scale optimization problems. Adding more agents to the swarm allows for better coverage of the solution space, which can be especially beneficial for high-dimensional optimization problems.

In this work, we apply swarm intelligence to the fault diagnosis problem in analog circuits. This section presents the two applied search techniques. The respective canonical algorithms are presented. First, in Section 6.1, we present the search technique based on the behavior of birds flocking. Then, in Section 6.2, we introduce the search method based on the behavior of bee colonies in the search for food. We specifically selected these techniques as they are suitable for multimodal function optimization, as is the case in this work.

### 6.1. Particle Swarm-Based Technique

The Particle Swarm Optimization technique (PSO) draws inspiration from the collective behavior of birds and fish. So, a swarm of particles explores the search space to find the optimal point of the specified objective function. Each particle has a position defined by coordinates for each dimension of the objective function and an individual velocity that is continually updated based on collective movement and individual experiences. As the particles move through the search space, they store the best position they have encountered so far, as well as the best position found by the entire swarm up to that point in the optimization process. The quality of the particles’ positions is determined by evaluating the objective function *f* that models the problem, and each position represents a potential solution that needs to be evaluated. The main steps of the PSO algorithm used in this work are presented in Algorithm 1.
**Algorithm 1** PSO’s main stepsSet: ω, $\phi_{1}$, $\phi_{2}$, *N*Generate *N* particles in the search space randomicallyObtain $P_{i_{best}}=f\left(x_{i}\right)$Select $S_{best}$ or $G_{best}=best\left(P_{i_{best}}\right)$ or $L_{best}=best\left(P_{i_{best(\eta_{i})}}\right)$, $\eta_{i}$ is the neighborhood of particle $x_{i}$*t*: = 1**while** (t≤T) and (solution not yet found) **do**   **for** each particle $x_{i}$ **do**       Calculate $v_{i}\left(t+1\right)=\omega \cdot v_{i}\left(t\right)+\phi_{1}\cdot r_{1}\cdot \left(P_{i_{best}}-x_{i}\left(t\right)\right)+\phi_{2}\cdot r_{2}\cdot \left(S_{i_{best}}-x_{i}\left(t\right)\right)$       Check velocity control       Calculate new position $x_{i}\left(t+1\right)=x_{i}\left(t\right)+v_{i}\left(t+1\right)$       Select $P_{i_{best}}$ and $S_{best}$   **end for**   Get $S_{best}$ among $P_{i_{best}}$ for all particle *i*   *t*: = *t* + 1**end while**Return best particle’s details

The algorithm allows adjusting several parameters based on the specific application, such as the number of particles *N*, the coefficients ω, $\phi_{1}$, $\phi_{2}$, the maximum number of iterations *T*, and the maximum velocity, as well as the neighborhood $\eta_{i}$ of particle *i*. Clearly, these parameters influence the behavior and efficiency of the PSO in finding the optimal solution in the given search space. PSO can be used with different swarm topology configurations. The main one is the star, where the neighborhood of particle *i* consists of all the particles of the swarm. In this case, the strategy is known as global best, and the swarm’s best solution is termed $G_{best}$. Another commonly used topology is the ring, wherein a particle’s neighborhood consists of another two predefined particles of the swarm. Furthermore, the neighborhood can also be defined by any other configuration, where the particle’s neighbors are more than 2 and less than the total number of particles in the swarm. When the neighborhood is distinct from global best, the strategy is called local best and the best solution is termed $L_{best}$. Note that $x_{i}$ is the current position of particle i, $P_{i}$ is its personal best, and for generalization purposes we use $S_{best}$ to denominate the best solution independently of the used topology configuration.

### 6.2. Bat Echolocation-Based Technique

The Bat Algorithm (BA) is proposed in [32] and is inspired by the echolocation behavior of microbats. The main rules devised for the algorithm are inspired by the behavior of microbats. Bats use echolocation to sense distance and distinguish between food/prey and background barriers. They usually fly randomly with a fixed minimum pulse frequency $f_{min}$, varying the wavelength λ and loudness $A_{0}$ to search for prey, adjusting λ or $f_{min}$ automatically based on the proximity to their target. They can also adjust the pulse emission rate r∈[0,1]. Although the loudness can vary, it is assumed that the loudness volume varies from a minimum value $A_{min}$ to a maximum value $A_{0}$. In its initial development, features that would increase the complexity of computational geometry, such as time delay estimation and three-dimensional topography, are avoided. Additionally, the frequency *f* varies within the range [$f_{min}$, $f_{max}$], which corresponds to a variation in the wavelength [$\lambda_{min}$, $\lambda_{max}$]. In the implementation, the range can be adjusted by modifying the wavelengths (or frequencies). The detectable range ($\lambda_{max}$) should be chosen to be comparable to the size of the search space and then decrease the loudness for smaller intervals. It is also possible to vary the frequency while keeping the wavelength λ fixed since wavelength and frequency are related due to the fact that λ×f is constant. Higher frequencies have shorter wavelengths and cover a shorter distance. For bats, typical ranges are a few meters. The pulse rate can simply be in the range [0,1], where 0 means no pulse and 1 means the maximum emission rate. The main steps of the BA algorithm used in this work are presented in Algorithm 2.
**Algorithm 2** BA’s main stepsSet β as an array of random numbers in [0, 1], ϵ a random number in [-1, 1],Set α, γ and *N*Generate a bat colony com *N* bats and velocity *v*Define the frequency pulse *f* for each batInitialize the pulse rate *r* and sonority *A* for each bat*t*: = 1**while** (t≤T) and (solution not yet found) **do**   Compute the quality of bat $f_{i}$   Generate solutions using $f_{i}=f_{min}+\left(f_{max}-f_{min}\right)\beta_{i}$   Update velocity $v_{i}$ using $v_{i}\left(t\right)=v_{i}^{t-1}+\left(x_{i}\left(t\right)-x_{best}\right)f_{i}$   Update position $x_{i}$ using $x_{i}\left(t\right)=x_{i}\left(t-1\right)+v_{i}\left(t\right)$   **if** $\xi >r_{i}$, where ξ is random number ∈[0,1] **then**       Select the best solution       Generate a local solution around the best solution   **end if**   Generate a randomically flying solution using $x_{i}\left(t\right)=x_{i}\left(t-1\right)+\epsilon A\left(t\right)$   Confine a bat within the search space   **if** $rand<A_{i}$ & $f\left(x_{i}\right)<f\left(x^{*}\right)$ **then**       Increase the pulse emission rate $r_{i}$ using $r_{i}\left(t+1\right)=A_{i}\left(0\right)\left[1-e^{\left(-\gamma t\right)}\right]$       Reduce the sonority $A_{i}$ using $A_{i}\left(t+1\right)=\alpha A_{i}\left(t\right)$       Accept new solutions   **end if**   Classify the bats and get the best one $x^{*}$   Select the best one $x^{*}$; *t*: = *t* + 1**end while**Return best bat’s details

## 7. Performance Evaluation

This section presents and analyzes the results obtained from the application of Particle Swarm Optimization (PSO) and the Bat Algorithm (BA) as techniques for fault diagnosis in analog circuits. First, we introduce the developed methodology for diagnosing faults in analog electronic circuits. Then, we define the metrics used to evaluate the performance of the explored optimization techniques. After that, we present and analyze the results obtained from experiments conducted with Tow–Thomas and Butterworth Biquad filters using the PSO technique, followed by the presentation and analysis of those yielded using the BA technique. Finally, we compare the performances of the optimization techniques.

### 7.1. Evaluation Methodology

The proposed methodology for fault diagnosis in analog circuits consists of, starting from any circuit with *K* components and *N* accessible nodes, performing simulations for each accessible node or combination of nodes, and considering all components of the circuit. Each simulation involves 100 optimization runs. For each circuit component, the optimization process evaluates whether there are no faults (NF) or not. If there are indications of faults, simulation $S_{A_{i}}$ provides the number of runs in which the optimization yielded values for the admittance $A_{i}$ outside the normal operating range.

So, *N* experiments are conducted for each individual node or combination. Each experiment deals with the NF case or a fault case $F_{A_{i}}$, which denotes a faulty component $A_{i}$. Each experiment allows testing the circuit *K* components. In each simulation, termed $S_{A_{i}}$, related to the test of component $A_{i}$, there are two possibilities: only component $A_{i}$ varies across the whole search space while the remaining K−1 components vary only within the operating range determined by the manufacturer, or both $A_{i}$ and $A_{j}$, where j≠i and component $A_{i}$ is being investigated, vary across the search space while the remaining K−2 components vary within the operating range determined by the manufacturer. Thus, the complete circuit test consists of N×(K+1)×K simulations. The search space of the implementation is defined with the minimum value of zero and the maximum value up to 20% higher than the ideal component value, as defined by Equation (52):

(52) $$p_{i,initial}=0,0\leq p_{i}\leq 1,2*p_{i,ideal},$$

where $p_{i}$ is the value corresponding to component $A_{i}$. Note that for the first case study, 216 experiments are performed via 21,600 optimizations, while for the second case study, 550 experiments are performed via 55,000 optimizations. Moreover, as we applied two different optimization meta-heuristics, the number of optimizations for each circuit is doubled.

Given the above explanation, Table 1 lists the 9 different scenarios and the respective measured voltage values at each accessible node of the first case study circuit. Recall that the components have ideal values of 10kΩ for the resistors and 10nF for the capacitors. In this configuration, the circuit behaves as a low-pass filter.

In Cases 2 to 9, only the indicated components are changed compared to the component values of Case 1. Since the circuit has three accessible nodes, each case has seven possible combinations: 3 considering a single point, 3 considering two combined points, and 1 considering the combination of all three accessible nodes. The considered situations regarding nodes and node combinations are presented in Table 2, wherein $T_{i/j}$ refers to the combination of nodes $T_{i}$ and $T_{j}$ and $T_{i/j/k}$ refers to the combination $T_{i}$, $T_{j}$, and $T_{k}$. The optimization results obtained for each case are available in Appendix A of [33].

Table 3 provides the 11 different scenarios and the respective measured voltage values at each accessible node of the second case study circuit. The components have ideal values of 10kΩ for the resistors and 10nF for the capacitors. In this configuration, the circuit also behaves as a low-pass filter.

In Cases 2 to 11, only the indicated components are changed compared to the values of Case 1. Since the circuit has five accessible nodes, there are 31 possible combinations: 5 considering a single node, 10 considering the combination of two nodes, 10 considering the combination of three nodes, 5 considering the combination of four nodes, and 1 considering the combination of all five accessible nodes. The considered situations regarding all node combinations are presented in Table 4. wherein, as before, $T_{i/j}$ refers to the combination of nodes $T_{i}$ and $T_{j}$ and $T_{i/j/k}$ refers to the combination $T_{i}$, $T_{j}$, and $T_{k}$, etc. The optimization results obtained for each case are available in Appendix B of [33].

For this work, the software tools SapWin4™ and Circuit Maker™ are used. SapWin™ is used to obtain the transfer function of the accessible nodes in the circuit. This software allows the circuit to be represented by identifying the input node and the accessible nodes for circuit analysis. Based on this information, SapWin4™ provides the corresponding transfer function for the indicated node. On the other hand, Circuit Maker™ is a circuit analysis software where output signals can be simulated based on a generic input signal. It allows the measurement of characteristics such as the amplitude of the output signal and the phase difference between the input and output signals of a specific accessible node. These measurements are used as metrics when transforming the fault diagnosis problem into an optimization problem. It is worth noting that for a circuit under test with more than one accessible node, the simulations are performed incrementally using the nodes in the objective function, both individually and in combination with another node.

The confusion matrix is calculated by the classification process and displays the distribution of simulations in terms of their classes. So, for each considered node combination, we obtain the numbers of executions that return a classification NF and $F_{A_{i}}$ for i=1,…,K, which indicate the quantity of executions that return a failure in component $A_{i}$. After that, considering the yielded confusion matrix, the performance metrics values are computed. So, for the confusion matrices for the circuits under study and for all node combinations listed in Table 2 for the first case study and Table 4 for the second one, we evaluate the performance metrics for all cases. The overall value of the metrics are defined as the arithmetic mean of the evaluated metrics for each $F_{A_{i}}$ of the studied node combinations. Hereafter, we define the considered performance metrics.

### 7.2. Evaluation Metrics

Most diagnostic tests produce multiple or continuous results. Grouping categories or applying a cutoff value is often used to classify results as positive or negative. This classification allows for the comparison between a diagnostic test and its reference standard, where True Positive (TP) denotes the number of executions that do not show faults (NF) or show faults in component $A_{i}$ in the respective simulation $S_{A_{i}}$, True Negative (TN) represents the number of executions that show faults for a faultless circuit or do not show faults in component $A_{i}$ in the respective simulation $S_{A_{i}}$, False Positive (FP) is the number of executions that show faults in components other than $A_{i}$ in the simulations $S_{A_{i}}$, and False Negative (FN) is the number of executions that do not show faults in the defective component in the simulations. These definitions allow us to calculate the performance metrics considered in this work, which are accuracy, precision, sensitivity, and specificity based on the outcomes of the diagnostic simulations. These metrics are defined as follows:

- Accuracy is a measure to quantify the level of agreement between an expected value and the number of correct outcomes obtained. It is defined by Equation (53):
  (53) $$A=100\times \frac{\mathrm{VP}+\mathrm{VN}}{\mathrm{VP}+\mathrm{VN}+\mathrm{FP}+\mathrm{FN}}.$$
- Precision measures the closeness between the obtained values through the repetition of the evaluation process. It is defined by Equation (54):
  (54) $$P=100\times \frac{\mathrm{VP}}{\mathrm{VP}+\mathrm{FP}}.$$
- Sensitivity, also known as recall, measures the ratio of correct positive predictions to the total number of positive instances. It is defined by Equation (55):
  (55) $$R=100\times \frac{\mathrm{VP}}{\mathrm{VP}+\mathrm{FN}}.$$
- Specificity measures the ratio of cases correctly classified as negative to the total number of cases without faults in a specific component different from the one being analyzed. It is defined by Equation (56):
  (56) $$S=100\times \frac{\mathrm{VN}}{\mathrm{VN}+\mathrm{FP}}.$$

### 7.3. PSO’s Performance Results

The PSO algorithm is implemented in Python without the use of specific libraries. To enable result comparison, all performed optimizations had fixed parameters, as shown in Table 5, where *K* represents the number of components in the circuit under analysis. Recall that Circuit 1 includes 8 components and Circuit 2 includes 10 components that may have faults. The inertia, cognitive, and social coefficients are set up according to [32].

#### 7.3.1. First Case Study

The confusion matrices for all the node combination listed in Table 2, as achieved when applying the PSO meta-heuristic for the first case study, are available in [33]. Table 6 shows the overall values of the considered performance metrics.

Here, we analyze the results obtained by the PSO meta-heuristic considering the test simulations performed for Circuit 1. It is noteworthy to point out that when using node $T_{1}$, the best results are obtained for the case without failures, regarding accuracy, sensitivity, and specificity: 96.83%, 98.89%, and 96.74%, respectively. Nonetheless, the case of failure in capacitor $C_{1}$ shows the best precision, with 63.05%. This is also the case when using node $T_{2}$ of the circuit. The NF case obtains the best results regarding accuracy, sensitivity, and specificity: 96.88%, 99.34%, and 96.78%, respectively, while the case of failure in capacitor $C_{1}$ obtains the best precision, with 64.38%. In addition, when node $T_{3}$ is used, the NF case exhibits the best results for accuracy, sensitivity, and specificity: 96.88%, 99.56%, and 96.76%, respectively, but the case of failure in capacitor $C_{1}$ obtains the best precision, with 65.73%. This is also the case when a combination of nodes $T_{13}$ are exploited. The NF case shows the best results for accuracy, sensitivity, and specificity: 97.66%, 100.00%, and 97.53%, respectively, and the case of failure in capacitor $C_{1}$ obtains the best precision, with 71.74%. However, when using the combination of nodes $T_{12}$, $T_{23}$, and $T_{123}$, the NF case is the best, classified with accuracy rates between 97.73% and 98.31%, precision rates between 71.00% and 79.88%, sensitivity rates between 99.31% and 99.82%, and specificity rates between 97.60% and 98.21%.

**Figure 9:**
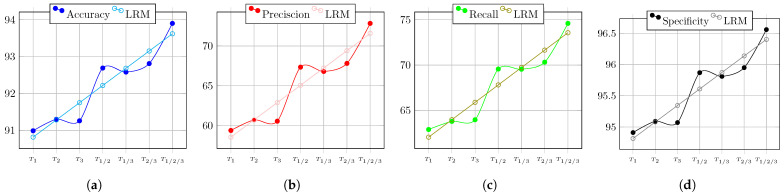
PSO’s performance for the first case study when varying the number of accessible nodes. (**a**) Accuracy; (**b**) Precision; (**c**) Recall; (**d**) Specificity.

The overall behaviors of the PSO’s performance during the test of Circuit 1, regarding the evaluation metrics, are depicted in Figure 9. Therein, the result of the linear regression model is also shown. It is clear that when more nodes are used, the circuit diagnostic result improves, becoming 3% more accurate, 10% more precise, 14% more sensitive, and almost 2% more specific.

#### 7.3.2. Second Case Study

The confusion matrices for all the node combination listed in Table 4, as achieved when applying the PSO meta-heuristic for the second case study, are available in [33]. Table 7 shows the overall values of the considered performance metrics for the combinations including 1 or 2 nodes.

In the sequel, we analyze the results obtained by the PSO meta-heuristic considering the test simulations performed for Circuit 2 based on a single node. It is noteworthy to point out that when using node $T_{1}$, the highest accuracy of 94.72% is achieved for the NF case, the best precision of 56.79% is achieved in the failure case of $C_{2}$, the highest sensitivity of 73.73% is yielded for the failure case in $C_{1}$, and the best specificity of 95.54% is obtained for the failure case in $R_{1}$. Similarly, when node $T_{2}$ is used, the highest accuracy of 95.37% is also achieved for the NF case, the best precision and highest specificity of 63.98% and 96.39%, respectively, are achieved in the failure case of $R_{6}$, and the highest sensitivity of 76.47% is also yielded for the failure case in $C_{1}$. However, when using node $T_{3}$, the NF case obtains the best results regarding accuracy, sensitivity, and specificity: 96.88%, 99.34%, and 96.78%, respectively. Nonetheless, the case of failure in capacitor $C_{1}$ shows the best precision with 63.05%. In addition, when node $T_{4}$ is used, the NF case exhibits the best results for accuracy, sensitivity, and specificity: 97.01%, 99.16%, and 96.95%, respectively. Meanwhile, the failure case in capacitor $C_{1}$ obtains the best precision with 60.23%. Analogically, when node $T_{5}$ is used, the NF case presents the highest results for accuracy, sensitivity, and specificity: 97.01%, 98.96%, and 96.96%, respectively. Nonetheless, the failure case in $R_{3}$ yields the best precision, with 60.35%.

Now, considering the test simulations performed for Circuit 2 based on a pair of nodes, it is noteworthy to point out that when using node $T_{1}$ and $T_{2}$, the case NF case achieves the highest accuracy and best sensitivity of 95.76% and 78.21%, respectively, while the failure case in $C_{3}$ obtains the best precision and the highest specificity of 63.18% and 96.33%, respectively. For the remaining case when combining node $T_{1}$ with the other nodes, the NF case is the winner with the highest accuracy, sensibility, and specificity of 97.79%, 100,00%, and 97.71%, respectively, when combined with $T_{3}$; 97.83%, 99.84%, and 97.75%, respectively, when combined with $T_{4}$; and 97,42%, 99,47% and 97,35%, respectively, when combined with $T_{5}$. Nonetheless, for those node combinations, the failure cases in $R_{5}$, $C_{1}$, and $R_{4}$ obtain the highest precision rates of 64.52%, 64.29%, and 63.22%, respectively. Moreover, when using combination $T_{2}$/$T_{3}$, the NF case achieves the highest results regarding accuracy, sensitivity, and specificity: 98.04%, 95.63%, and 98.16%, respectively. The failure case in capacitor $R_{5}$ shows the best precision, with 72.75%. For the combinations of node $T_{2}$ with either $T_{4}$ or $T_{5}$, the NF case yields the best rates regarding all four metrics, with 97.91%, 73.00%, 92.64%, and 98.19% and 97.97%, 69.90%, 96.15%, and 98.06%, respectively. For the combinations $T_{34}$, $T_{35}$, and $T_{45}$, the NF case also obtains the best rates regarding accuracy, sensitivity, and specificity: 97.47%, 99.83%, and 97.39%; 97.34%, 89.80%, and 97.68%; and 97.39%, 99.46%, and 97.32%, respectively. In the three combinations, the failure case in $R_{4}$ obtains the highest precision with 64.97%, 69.41%, and 64.78%, respectively.

Table 8 shows the overall values of the performance metrics for the combinations including 3 nodes.

We now analyze the results obtained by the PSO meta-heuristic considering the test simulations performed for Circuit 2 based on node triplets. It is noteworthy to point out that when using combinations $T_{123}$, $T_{124}$, $T_{234}$, and $T_{235}$, the NF case achieves the best rates regarding all four metrics, with 98.24%, 72.80%, 98.78%, and 98.21%; 97.75%, 73.00%, 89.24%, and 98.21%; 98.32%, 75.60%, 96.80%, and 98.40%; and 98.38%, 74.60%, 98.94%, and 98,35%, respectively. For the node combinations $T_{125}$ and $T_{134}$, the NF case obtains the highest results regarding accuracy, sensitivity, and specificity: 98.01%, 98.29%, and 97.99% and 98.01%, 99.85%, and 97.93%, respectively. For both combinations, the failure case in capacitor $R_{7}$ shows the best precision with 69.65% and 68.69%. This performance is repeated for the node combinations $T_{135}$, $T_{245}$, and $T_{345}$, with the rates 98.12%, 99.57%, and 98.05%; 98.18%, 97.47%, and 98.21%; and 97.74%, 100.00%, and 97.64% for accuracy, sensitivity, and specificity, respectively. Nonetheless, for these combinations, the highest precision is achieved for the failure case in $R_{2}$, with 70.11%, $C_{2}$, with 3.31%, and $R_{4}$, with 68.64%, respectively.

Table 9 shows the values of the metrics for the combinations including 4 or 5 nodes.

In the sequel, we analyze the results obtained by the PSO meta-heuristic considering the test simulations performed for Circuit 2 based on 4 and 5 nodes. It is noteworthy to point out that when using combinations $T_{1}$–$T_{3}$/$T_{5}$, $T_{1}$/$T_{2}$/$T_{4}$/$T_{5}$, and $T_{2}$–$T_{5}$, again the NF case achieves the highest rates regarding all four metrics, with 98.39%, 74.70%, 100%, and 98.31%; 98.38%, 74.90%, 99.21%, and 98.34%; and 98.50%, 76.10%, 98.96%, and 98.48%, respectively. For the combination $T_{1345}$, the NF case obtains the best rates regarding accuracy, sensitivity, and specificity, with 97.99%, 100%, and 97.90%, respectively. However, the failure case in $R_{7}$ obtains the highest precision, with 72.16%. Last but not least, the combination that includes all 5 accessible nodes of Circuit 2 allows the highest rates with respect to all metrics, with 98.43%, 76.00%, 99.61%, and 98.37%, respectively, for accuracy, precision, sensitivity, and specificity.

**Figure 10:**
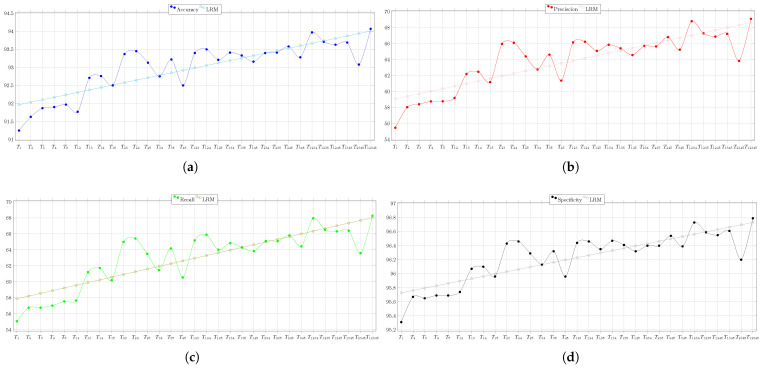
PSO’s performance for the second case study when varying the number of accessible nodes. (**a**) Accuracy; (**b**) Precision; (**c**) Recall; (**d**) Specificity.

The overall behaviors of the PSO’s performance during the test of Circuit 2, regarding the evaluation metrics, are depicted in Figure 10. Therein, the result of the linear regression model is also shown. It is clear that when more nodes are used, the circuit diagnostic result improves, becoming 3% more accurate, 14% more precise, 13% more sensitive, and almost 1.5% more specific.

### 7.4. BA’s Performance Results

The BA is implemented in Python without the use of specific libraries. To enable result comparison, all performed optimizations had fixed parameters, as shown in Table 10, where *K* represents the number of components in the circuit under analysis. Recall that Circuit 1 includes 8 components, and Circuit 2 includes 10 components that may have faults. This algorithm uses either wavelength or frequency as implementation parameters. The higher the frequency used, the shorter the wavelength, resulting in shorter distances to be traveled to find the optimal location.

#### 7.4.1. First Case Study

The confusion matrices for all the node combination listed in Table 2, as achieved when applying the BA meta-heuristic for the first case study, are available in [33]. Table 11 shows the overall values of the considered performance metrics.

Here, we analyze the results obtained by the BA meta-heuristic considering the test simulations performed for Circuit 1. It is noteworthy to point out that, as for PSO, when using node $T_{1}$, the best results are obtained for the NF case, regarding accuracy, sensitivity, and specificity: 96.91%, 99.58%, and 96.79%, respectively. Nonetheless, the case of failure in capacitor $C_{1}$ shows the best precision, with 69.70%. This is also the case when using node $T_{2}$ of the circuit. The NF case obtains the best results regarding accuracy, sensitivity, and specificity: 97.17%, 99.22%, and 97.06%, respectively, while the case of failure in capacitor $C_{1}$ obtains the best precision, with 68.56%. Again, when node $T_{3}$ is used, the NF case exhibits the best results for accuracy, sensitivity, and specificity: 97.01%, 98.62%, and 96.93%, respectively, but the case of failure in capacitor $C_{1}$ obtains the best precision, with 69.96%. For the case when node combinations $T_{12}$, $T_{23}$, and $T_{123}$ are exploited, the NF case shows the best results for all four metrics i.e., accuracy, precision, sensitivity, and specificity: 97.89%, 75.13%, 99.50%, and 97.78%; 98.05%, 77.50%, 99.84%, and 97.92%; and 98.73%, 87.50%, 98.45%, and 98.75%, respectively. However, when using the combination $T_{13}$, the NF case is the best classified only regarding accuracy, sensitivity, and specificity: 97.83%, 99.67%, and 97.71%, respectively. The failure case in capacitor $C_{1}$ shows the highest precision, with 75.55%.

**Figure 11:**
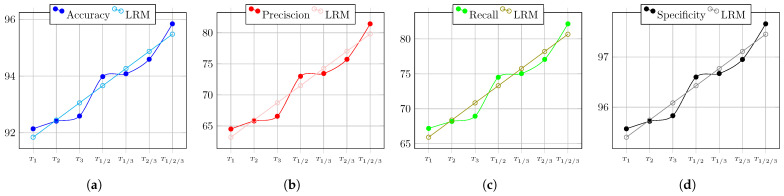
BA’s performance for the first case study when varying the number of accessible nodes. (**a**) Accuracy; (**b**) Precision; (**c**) Recall; (**d**) Specificity.

The overall behaviors of the BA’s performance during the test of Circuit 1 regarding the evaluation metrics are depicted in Figure 11. Therein, the result of the linear regression model is also shown. It is clear that when more nodes are used, the circuit diagnostic result improves, becoming 4% more accurate, 16% more precise, 17% more sensitive, and almost 2% more specific.

#### 7.4.2. Second Case Study

The confusion matrices for all the node combination listed in Table 4, as achieved when applying the BA meta-heuristic for the second case study, are available in [33]. Table 12 shows the overall values of the considered performance metrics for the combinations including 1 or 2 nodes.

In the sequel, we analyze the results obtained by the BA meta-heuristic considering the test simulations performed for Circuit 2 based on a single node. It is noteworthy to point out that when using node $T_{1}$, BA obtained the same results as the PSO. That is, the highest accuracy of 94.72% is achieved for the NF case, the best precision of 56.79% is achieved in the failure case of $C_{2}$, the highest sensitivity of 73.73% is yielded for the failure case in $C_{1}$, and the best specificity of 95.54% is obtained for the failure case in $R_{1}$. Similarly, when node $T_{2}$ is used, BA achieved the same results as PSO. That is, the highest accuracy of 95.37% is achieved for the NF case, the best precision and highest specificity of 63.98% and 96.39%, respectively, are achieved in the failure case in $R_{6}$, and the highest sensitivity of 76.47% is yielded for the failure case in $C_{1}$. However, when using node $T_{3}$, BA reaches different results. The NF case obtains the best results regarding accuracy and sensitivity, with 97.02% and 98.44%, respectively. The case of failure in $R_{6}$ shows the best precision and specificity, with 68.97% and 97.04%, respectively. This is also the case when node $T_{4}$ is used. The NF case exhibits the best results for accuracy and sensitivity only, with 97.21% and 98.72%, respectively, while the failure case in $R_{6}$ obtains the best precision and specificity, with 70.72% and 97.25%, respectively. In contrast, when node $T_{5}$ is used, the NF case presents the highest results for accuracy, sensitivity, and specificity, with 97.26%, 98.41%, and 97.22%, respectively. Nonetheless, the failure case in $R_{6}$ yields the best precision. with 68.63%.

Now, considering the test simulations performed for Circuit 2 based on a pair of nodes, it is noteworthy to point out that for all the node pairs with $T_{1}$, BA yields the same best results as PSO when using combination $T_{12}$. This is also the case for the pairs with $T_{2}$ Moreover, when using combination $T_{23}$, the NF case achieves the highest results regarding accuracy, sensitivity, and specificity: 98.04%, 95.63%, and 98.16%, respectively. The failure case in capacitor $R_{5}$ shows the best precision, with 72.75%. For the combinations of node $T_{2}$ with either $T_{4}$ or $T_{5}$, the NF case yields the best rates regarding all four metrics, with 97.91%, 73.00%, 92.64%, and 98.19% and 97.97%, 69.90%, 96.15%, and 98.06%, respectively. For the combinations $T_{34}$ and $T_{35}$, the NF case also obtains the best rates regarding accuracy, sensitivity, and specificity: 97.49%, 99.18%, and 97.42%; 96.94%, 88.24%, and 97.34%; and 97.39%, 99.46%, and 97.32%, respectively. In both combinations, the failure case in $R_{6}$ obtains the highest precision, with 72.18% and 72.50%, respectively. In contrast, for the node combination $T_{45}$, the NF case achieves the best rates regarding accuracy and sensitivity only, with 97.42% and 98.07%, respectively. The failure case in $R_{6}$ obtains the highest rate regarding precision and specificity, with 73.74% and 97.48%, respectively.

Table 13 shows the overall values of the performance metrics for the combinations including 3 nodes.

We now analyze the results obtained by the BA meta-heuristic considering the test simulations performed for Circuit 2 based on node triplets. It is noteworthy to point out that BA achieves the same best metric values for the same cases, i.e., when using combinations $T_{123}$, $T_{124}$, $T_{234}$, and $T_{235}$. This is also the case for node combinations $T_{125}$ and $T_{134}$. For node combinations $T_{135}$, $T_{145}$, $T_{245}$, and $T_{345}$, the NF case yields the best rates for accuracy, sensitivity, and specificity, with 97.65%, 99.24%, and 97.58%; 97.91%, 99.71%, and 97.83%; and 97.87%, 99.85%, and 97.77%, respectively. Nonetheless, for these combinations, the highest precision is achieved for the failure case in $R_{1}$ for the first combination, with 74.11 while it is achieved for failure in $R_{6}$ for the last three combination with a precision of 75.34% for the first one and 75.34% for the last two.

Table 14 shows the values of the metrics for the combinations including 4 or 5 nodes.

In the sequel, we analyze the results obtained by the PSO meta-heuristic considering the test simulations performed for Circuit 2 based on 4 and 5 nodes. It is noteworthy to point out that when using combinations $T_{1235}$, the NF case obtains the best rates regarding accuracy, sensitivity, and specificity, with 98.42%, 98.98%, and 98.38%, respectively. The highest precision is achieved for the failure case in $R_{7}$, with a value of 79.71%. All remaining node combinations, including the one with all five nodes, were allowed to reach the best performance for the NF case regarding all four metrics, with node combination $T_{1234}$ having 98.55%, 78.10%, 99.24%, and 98.51%, respectively; $T_{1235}$ obtains 98.39%, 74.70%, 100%, and 98.31%, respectively; $T_{1245}$ obtains 98.38%, 74.90%, 99.21%, and 98.34%, respectively; $T_{2345}$ has 98.71%, 82.00%, 99.51%, and 98.66%, respectively; and the five-node combination $T_{12345}$ achieves 99.05%, 87.40%, 99.43%, and 99.03%, respectively. It is worth noting that this last case is the best in all the investigation.

**Figure 12:**
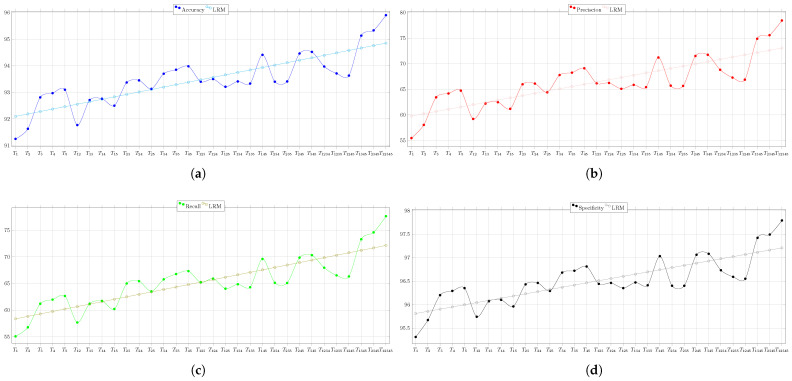
BA’s performance for the second case study when varying the number of accessible nodes. (**a**) Accuracy; (**b**) Precision; (**c**) Recall; (**d**) Specificity.

The overall behaviors of the BA’s performance during the test of Circuit 2 regarding the evaluation metrics are depicted in Figure 12. Therein, the result of the linear regression model is also shown. It is clear that when more nodes are used, the circuit diagnostic result improves, becoming 5% more accurate, 24% more precise, 24% more sensitive, and almost 3% more specific.

### 7.5. Performance Comparison: PSO vs. BA

The performances of PSO and BA are evaluated based on the values of the defined metrics and comparing the two case studies developed in this work. It is worth noting that the parameters used for the compared techniques in the two studied circuits are the same, differing only in the number of possible components with failures, where the first circuit has 8 components, the second has 10, and the number of accessible nodes, with 3 nodes for the first circuit and 5 nodes for the second. Table 15 presents the global evaluation metrics in relation to the increment of the use of accessible nodes in the circuits, using Table 2 and Table 4 as the basis. The values are arithmetic averages of the metric’s values considering the number of nodes used in all cases.

The overall improvements regarding the metrics as achieved by PSO vs. BA are presented in Table 16. Globally, it can be observed that with the incremental node usage through node combinations, there is an increase in the evaluation metrics for both optimization techniques. For PSO application, the increase highlight is in precision and sensitivity for both circuits.

For Circuit 1, when using three accessible nodes, the precision increases by 12.63% compared to the experiment using an individual node. Similarly, for sensitivity, there is an increase of 11.06%. For Circuit 2, the precision increases by 11.22% when analyzing the five-node combination compared to an individual node. As for sensitivity, there is an increase of 11.65%. For the BA application, in the case of Circuit 1, the precision metric increased by 15.82% when comparing the experiment using three nodes with the experiment using an individual node. In terms of sensitivity, there is an increase of 14.05%. For Circuit 2, precision increases by 17.27% when analyzing the combination of the five nodes compared to an individual node. As for sensitivity, there is an increase of 18.11%.

**Figure 13:**
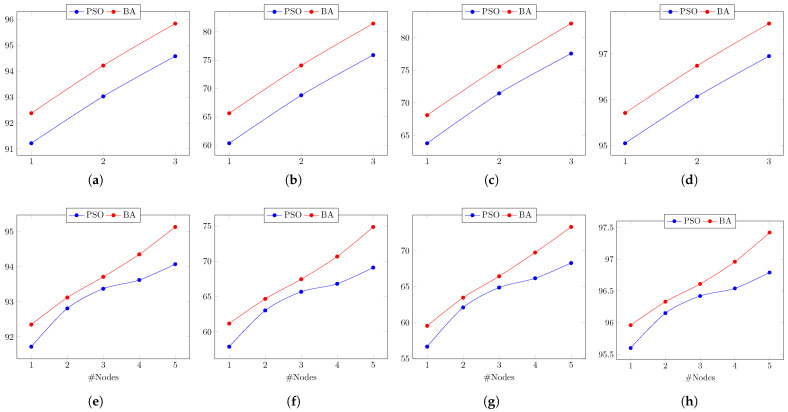
Comparison of the evaluation metrics regarding the testing of circuits as achieved by PSO vs. BA. (**a**) Accuracy for Circuit 1; (**b**) Precision for Circuit 1; (**c**) Recall for Circuit 1; (**d**) Specificity for Circuit 1; (**e**) Accuracy for Circuit 2; (**f**) Precision for Circuit 2; (**g**) Recall for Circuit 2; (**h**) Specificity for Circuit 2.

Figure 13 shows the results presented in Table 15. In Figure 13a–d, the global performance achieved by PSO and BA for Circuit 1 are graphically presented, allowing the analysis of the impact of the number of nodes considered on the performance of the optimization techniques.

It can be observed that the best performance in Circuit 1 for the considered metrics is achieved when using BA with the combination of three nodes. Furthermore, in Figure 13e–h, the global performance achieved by PSO and BA for Circuit 2 are graphically presented, allowing the analysis of the impact of the number of nodes considered on the performance of the optimization techniques. Once again, the previous observation consolidates the conclusion that the best performance in Circuit 2 for the considered metrics is achieved when using BA with the combination of three nodes.

The average execution times are recorded for the execution of the optimization process for each of the circuits, with and without failures. Table 17 presents the execution times for PSO and BA with respect to the number of nodes used in the two case studies, for cases without failure (NF) and with failure (WF).

**Figure 14:**
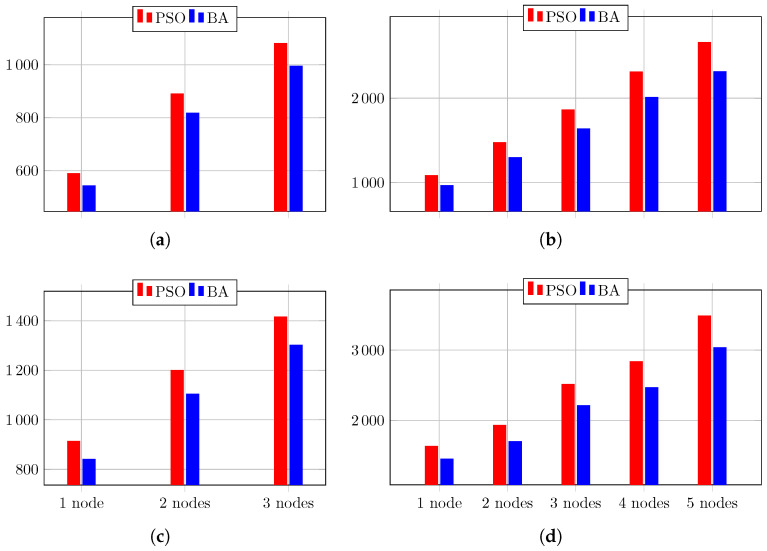
Comparison of the execution times(*s*) regarding the testing of circuits as required by PSO vs. BA. (**a**) Faultless Circuit 1; (**b**) Faultless Circuit 2; (**c**) Faulty Circuit 1; (**d**) Faulty Circuit 2.

Figure 14 presents a comparison of the execution times of the implementations for the case studies, considering cases without failure and with failure. It can be observed that the execution times in Circuit 2 are higher compared to Circuit 1. This is due to the increased complexity of Circuit 2 compared to that of Circuit 1. Circuit 2 has more components, is a third-order filter, and has a higher mathematical complexity in its transfer function. It can also be noted that, in both circuits, the BA’s execution time is shorter than the PSO’s.

### 7.6. Performance Comparison: Optimization vs. Classification

Table 18 establish a comparative analysis based on the accuracy rates achieved by eight works about fault diagnosis of analog circuit. The results regard the Biquad filter. The considered scenarios are those regarding faultless circuits and faulty circuits in components $R_{1}$–$R_{4}$, $C_{1}$, and $C_{2}$. The average accuracy rates are presented.

These works are mainly based on the classification approach using different types of learning process, either supervised or unsupervised. The proposed approach based on PSO for fault diagnosis achieves better results in two cases (1 and 3), but obtains worse results in the remaining six cases (2, 4–7). On the other hand, the proposed approach based on BA is more successful. It allows one to detect faults with higher or similar accuracy rates when compared to the those based on the classification approach. As for the PSO-based approach, the BA is better in two cases (1 and 3), but worse in one case (4). Nonetheless, in the remaining five cases, it shows similar performances. As concluded before, the BA-based fault diagnosis performs better than the PSO-based approach. That said, it is worth noting that any fault diagnosis method that is based on the classification approach would require a massive dataset to be used in the learning stage, which in some case could be inconvenient. Needless to say, any fault diagnosis method that is based on the optimization approach is completely free of such requirement. Only a few measurements, i.e, at every observable node, are required to launch the optimization process, as opposed to a few thousands of samples of the training, validation, and test datasets required by machine learning-based approaches.

## 8. Conclusions

This work addresses the problem of fault diagnosis in analog electronic circuits through the implementation of two optimization techniques: PSO and BA. Two case studies are conducted for each optimization technique used. In the first case study, the Tow–Thomas Biquad circuit is utilized, which is a second-order filter. In the second case study, the Butterworth filter is used. It is a third-order filter.

The purpose of using optimization techniques is to find the parameters of the circuit’s components given the transfer functions of the accessible nodes and verify if the optimum value found corresponds to the correct operating values of the circuit. The first step of the implementations is to verify whether there is a fault or not. The second step consists of identifying the possible faulty components. Based on the obtained results, we can safely conclude that, in both techniques, it is possible to identify the defective components in most cases or at least reduce the number of possible faulty components by 75% in the remaining ones.

In both case studies, the best performance of the metrics is achieved when analyzing the combination of the all accessible nodes of the circuit. So, it is safe to conclude that the totality of the accessible nodes must be used to reach the most reliable circuit diagnostic. Moreover, a higher performance is observed in both circuits when using the BA technique to the detriment of the PSO method. Additionally, in both cases, the BA technique also requires less execution time to reach the mentioned results. Hence, the BA optimization technique is more adequate to the application of analog circuit diagnostic, providing better performance with a reduced execution time. In additiob, it is worth noting that the optimization-based methodology using BA achieved similar performance to machine learning and classification-based methodologies.

With this work, we have consolidated the conclusion that considering more observable nodes, the proposed approach achives higher accuracy. So, when applying this methodology to complex circuit diagnostic, the idea is to study only the case regarding the combination of all available nodes for observation in the considered circuit. We can safely conclude that the proposed approach scales well with the circuit complexity.

As a future work, the application of other optimization techniques could be explored to seek performance improvements and further reduce the execution time of the implementations. By applying other bio-inspired techniques or mathematical models, better results may be achieved. Furthermore, the study of new circuit analysis techniques to enhance the system’s constraints and improve the performance of the proposed methodology is another avenue for exploration, specifically, using circuit sensitivity analysis to identify the influence of each component on the accessible nodes. This should help detect variations beyond the predefined operating range set by component manufacturers. Increasing the constraints in the system’s model may influence the search for multiple faults in the circuits.

## Figures and Tables

**Table 1:** Measured voltage at nodes $T_{1}$–$T_{3}$ in Tow–Thomas Biquad filter.

Scenarios	Fault	Value	$T_{1}$ (V)	$T_{2}$ (V)	$T_{3}$ (V)
Case 1	NF	-	−0.283−j0.451	0.717−j0.451	0.717−j0.451
Case 2	$R_{1}$	4.32kΩ	−0.655−j1.043	1.659−j1.043	1.659−j1.043
Case 3	$R_{2}$	6.5kΩ	−0.314−j0.325	0.517−j0.499	0.517−j0.499
Case 4	$R_{3}$	4.3kΩ	−0.068−j0.252	0.401−j0.108	0.401−j0.108
Case 5	$R_{4}$	5.0kΩ	−0.089−j0.286	0.910−j0.286	0.910−j0.286
Case 6	$R_{5}$	6.0kΩ	−0.523−j0.499	0.795−j0.832	0.477−j0.499
Case 7	$R_{6}$	4.0kΩ	−0.059−j0.236	0.376−j0.095	0.941−j0.236
Case 8	$C_{1}$	5nF	−0.283−j0.450	0.717−j0.450	0.717−j0.450
Case 9	$C_{2}$	4nF	−0.059−j0.236	0.941−j0.236	0.941−j0.236

**Table 2:** Used node combinations for the scenarios regarding the test of Tow–Thomas Biquad filter.

#Nodes	Node Combinations
1	$T_{1}$, $T_{2}$, $T_{3}$
2	$T_{1/2}$, $T_{1/3}$, $T_{1/2}$
3	$T_{1/2/3}$

**Table 3:** Measured voltage at nodes $T_{1}$–$T_{5}$ in Butterworth filter.

Scenarios	Fault	Value	$T_{1}$ (V)	$T_{2}$ (V)	$T_{3}$ (V)	$T_{4}$ (V)	$T_{5}$ (V)
Case 1	NF	0.7170−j0.4505	1.4339−j0.9009	1.5904−j1.6511	0.3965−j1.9003	0.7929−j3.8005	
Case 2	$R_{1}$	5.0kΩ	0.9102−j0.2859	1.8203−j0.5719	2.2054−j1.3447	0.9754−j1.9576	1.9509−j3.9152
Case 3	$R_{2}$	4.0kΩ	0.7170−j0.4505	2.5093−j1.5767	2.7833−j2.8895	0.6938−j3.3254	1.3876−j6.6509
Case 4	$R_{3}$	6.0kΩ	0.7170−j0.4505	1.1471−j0.7208	1.2723−j1.3209	0.3172−j1.5202	0.6344−j3.0404
Case 5	$R_{4}$	5.0kΩ	0.7170−j0.4505	1.4339−j0.9010	1.5450−j1.2095	0.5629−j1.5632	1.1257−j3.1263
Case 6	$R_{5}$	6.0kΩ	0.7170−j0.4505	1.4339−j0.9010	1.6806−j1.3025	1.0416−j1.6952	2.0832−j3.3903
Case 7	$R_{6}$	4.0kΩ	0.7170−j0.4505	1.4339−j0.9010	2.6032+j1.3513	2.4751−j0.2039	8.6628−j0.7135
Case 8	$R_{7}$	5.0kΩ	0.7170−j0.4505	1.4339−j0.9010	0.9648−j1.5025	0.0149−j1.5119	0.0224−j2.2678
Case 9	$C_{1}$	4nF	0.9406−j0.2364	1.8812−j0.4728	2.3177−j1.2343	1.1056−j1.929	2.2112−j3.8580
Case 10	$C_{2}$	5nF	0.7170−j0.4505	1.4339−j0.9010	1.0475−j1.2300	0.1969−j1.3537	0.3938−j2.7075
Case 11	$C_{3}$	4nF	0.7170−j0.4505	1.4339−j0.9010	2.0225−j0.3401	1.8219−j0.7981	3.6438−j1.5961

**Table 4:** Used node combination in the scenarios regarding the test of Butterworth filter.

#Nodes	Node Combinations
1	$T_{1}$, $T_{2}$, $T_{3}$, $T_{4}$, $T_{5}$
2	$T_{1/2}$, $T_{1/3}$, $T_{1/4}$, $T_{1/5}$, $T_{2/3}$, $T_{2/4}$, $T_{2/5}$, $T_{3/4}$, $T_{3/5}$, $T_{4/5}$
3	$T_{1/2/3}$, $T_{1/2/4}$, $T_{1/2/5}$, $T_{1/3/4}$, $T_{1/3/5}$, $T_{1/4/5}$, $T_{2/3/4}$, $T_{2/3/5}$, $T_{2/4/5}$, $T_{3/4/5}$
4	$T_{1/2/3/4}$, $T_{1/2/3/5}$, $T_{1/2/4/5}$, $T_{1/3/4/5}$, $T_{2/3/4/5}$
5	$T_{1/2/3/4/5}$

**Table 5:** PSO’s parameter settings.

Parameter	Value
#Dimensions	K
#Iterations	1000
#Particles	30
ω	0.5
$\phi_{1}$	1.49
$\phi_{2}$	1.49
$f_{min}$	0.0
Error	$10^{-15}$

**Table 6:** PSO’s achieved results for Circuit 1 regarding the defined metrics and the listed scenarios.

Metric (%)	$T_{1}$	$T_{2}$	$T_{3}$	$T_{1/2}$	$T_{1/3}$	$T_{2/3}$	$T_{1/2/3}$
A	90.99	91.30	91.26	92.69	92.58	92.81	93.90
P	59.38	60.70	60.55	67.34	66.80	67.81	72.84
R	62.89	63.78	63.96	69.56	69.54	70.32	74.60
S	94.91	95.09	95.07	95.87	95.81	95.95	96.56

**Table 7:** PSO’s achieved results for Circuit 2 regarding the defined metrics and the listed scenarios with 1 and 2 node combinations.

Metric (%)	$T_{1}$	$T_{2}$	$T_{3}$	$T_{4}$	$T_{5}$	$T_{1/2}$	$T_{1/3}$	$T_{1/4}$	$T_{1/5}$	$T_{2/3}$	$T_{2/4}$	$T_{2/5}$	$T_{3/4}$	$T_{3/5}$	$T_{4/5}$
A	91.25	91.63	91.87	91.90	91.97	91.77	92.71	92.76	92.50	93.37	93.45	93.13	92.75	93.22	92.50
P	55.46	58.04	58.41	58.76	58.78	59.20	62.20	62.48	61.18	65.97	66.11	64.40	62.76	64.62	61.37
R	55.06	56.76	56.76	57.02	57.55	57.66	61.20	61.73	60.20	65.00	65.43	63.51	61.46	64.18	60.54
S	95.31	95.67	95.65	95.69	95.69	95.74	96.07	96.10	95.96	96.43	96.46	96.29	96.13	96.32	95.96

**Table 8:** PSO’s achieved results for Circuit 2 regarding the defined metrics and the listed scenarios with 3 node combinations.

Metric (%)	$T_{1/2/3}$	$T_{1/2/4}$	$T_{1/2/5}$	$T_{1/3/4}$	$T_{1/3/5}$	$T_{1/4/5}$	$T_{2/3/4}$	$T_{2/3/5}$	$T_{2/4/5}$	$T_{3/4/5}$
A	93.40	93.50	93.21	93.41	93.33	93.16	93.40	93.41	93.58	93.28
P	66.16	66.24	65.09	65.86	65.41	64.57	65.71	65.65	66.82	65.24
R	65.19	65.91	64.02	64.85	64.29	63.84	65.12	65.09	65.83	64.44
S	96.44	96.46	96.35	96.47	96.41	96.32	96.40	96.40	96.54	96.39

**Table 9:** PSO’s achieved results for Circuit 2 regarding the defined metrics and the listed scenarios with 4 and 5 node combinations.

Metric (%)	$T_{1/2/3/4}$	$T_{1/2/3/5}$	$T_{1/2/4/5}$	$T_{1/3/4/5}$	$T_{2/3/4/5}$	$T_{1/2/3/4/5}$
A	93.97	93.71	93.63	93.69	93.08	94.07
P	68.81	67.29	66.88	67.23	63.84	69.11
R	67.96	66.52	66.32	66.41	63.59	68.28
S	96.73	96.59	96.55	96.61	96.20	96.79

**Table 10:** BA’s parameter settings.

Parameter	Value
#Dimensions	K
#Iterations	1000
#Bats	30
Alfa	0.5
Beta	0.5
Initial pulse rate	0.1
$f_{min}$	0 Hz
$f_{max}$	500 kHz

**Table 11:** BA’s achieved results for Circuit 1 regarding the defined metrics and the listed scenarios.

Metric (%)	$T_{1}$	$T_{2}$	$T_{3}$	$T_{1/2}$	$T_{1/3}$	$T_{2/3}$	$T_{1/2/3}$
A	92.14	92.41	92.59	93.98	94.08	94.59	95.84
P	64.51	65.81	66.57	73.00	73.45	75.74	81.45
R	67.19	68.19	68.94	74.51	75.04	77.07	82.16
S	95.57	95.72	95.83	96.60	96.67	96.95	97.66

**Table 12:** BA’s achieved results for Circuit 2 regarding the defined metrics and the listed scenarios with 1 and 2 node combinations.

Metric (%)	$T_{1}$	$T_{2}$	$T_{3}$	$T_{4}$	$T_{5}$	$T_{1/2}$	$T_{1/3}$	$T_{1/4}$	$T_{1/5}$	$T_{2/3}$	$T_{2/4}$	$T_{2/5}$	$T_{3/4}$	$T_{3/5}$	$T_{4/5}$
A	91.25	91.63	92.81	92.97	93.10	91.77	92.71	92.76	92.50	93.37	93.45	93.13	93.70	93.85	93.98
P	55.46	58.04	63.43	64.18	64.73	59.20	62.20	62.48	61.18	65.97	66.11	64.40	67.78	68.25	69.10
R	55.06	56.76	61.19	61.97	62.65	57.66	61.20	61.73	60.20	65.00	65.43	63.51	65.77	66.77	67.32
S	95.31	95.67	96.20	96.29	96.35	95.74	96.07	96.10	95.96	96.43	96.46	96.29	96.68	96.72	96.81

**Table 13:** BA’s achieved results for Circuit 2 regarding the defined metrics and the listed scenarios with 3 node combinations.

Metric (%)	$T_{1/2/3}$	$T_{1/2/4}$	$T_{1/2/5}$	$T_{1/3/4}$	$T_{1/3/5}$	$T_{1/4/5}$	$T_{2/3/4}$	$T_{2/3/5}$	$T_{2/4/5}$	$T_{3/4/5}$
A	93.40	93.50	93.21	93.41	93.33	94.41	93.40	93.41	94.46	94.52
P	66.16	66.24	65.09	65.86	65.41	71.21	65.71	65.65	71.51	71.74
R	65.19	65.91	64.02	64.85	64.29	69.61	65.12	65.09	69.86	70.32
S	96.44	96.46	96.35	96.47	96.41	97.03	96.40	96.40	97.06	97.08

**Table 14:** BA’s achieved results for Circuit 2 regarding the defined metrics and the listed scenarios with 4 and 5 node combinations.

Metric (%)	$T_{1/2/3/4}$	$T_{1/2/3/5}$	$T_{1/2/4/5}$	$T_{1/3/4/5}$	$T_{2/3/4/5}$	$T_{1/2/3/4/5}$
A	93.97	93.71	93.63	95.13	95.33	95.90
P	68.81	67.29	66.88	74.87	75.57	78.44
R	67.96	66.52	66.32	73.30	74.60	77.64
S	96.73	96.59	96.55	97.42	97.49	97.79

**Table 15:** Evaluation metrics as achieved by the PSO and BA meta-heuristics.

		#Nodes									
**Metrics (%)**		**1**		**2**		**3**		**4**		**5**	
		**PSO**	**BA**	**PSO**	**BA**	**PSO**	**BA**	**PSO**	**BA**	**PSO**	**BA**
Circuit 1	A	91.18	92.38	92.69	94.22	93.90	95.84	-	-	-	-
	P	60.21	65.63	67.32	74.06	72.84	81.45	-	-	-	-
	S	63.54	68.11	69.80	75.54	74.60	82.16	-	-	-	-
	E	95.02	95.71	95.88	96.74	96.56	97.66	-	-	-	-
Circuit 2	A	91.72	92.35	92.81	93.12	93.37	93.71	93.62	94.35	94.07	95.90
	P	57.89	61.17	63.03	64.67	65.68	67.46	66.81	70.68	69.11	78.44
	S	56.63	59.53	62.09	63.46	64.86	66.43	66.16	69.74	68.28	77.64
	E	95.60	95.96	96.15	96.33	96.42	96.61	96.54	96.96	96.79	97.79

**Table 16:** Improvement comparison when varying the number of considered nodes.

		#Nodes							
**Improvement (%)**		**2**		**3**		**4**		**5**	
		**PSO**	**BA**	**PSO**	**BA**	**PSO**	**BA**	**PSO**	**BA**
Circuit 1	A	1.51	1.84	2.72	3.46	-	-	-	-
	P	7.11	8.43	12.63	15.82	-	-	-	-
	R	6.26	7.43	11.06	14.05	-	-	-	-
	S	0.86	1.03	1.54	1.95	-	-	-	-
Circuit 2	A	1.09	0.77	1.65	1.36	1.90	2.00	2.35	3.55
	P	5.14	3.50	7.79	6.29	8.92	9.51	11.22	17.27
	R	5.46	3.93	8.23	6.90	9.53	10.21	11.65	18.11
	S	0.55	0.37	0.82	0.65	0.94	1.00	1.19	1.83

**Table 17:** Execution times regarding the tested of circuits as achieved by PSO vs. BA.

#Nodes	Time (s)							
	**Circuit 1**				**Circuit 2**			
	**NF**		**WF**		**NF**		**WF**	
	**PSO**	**BA**	**PSO**	**BA**	**PSO**	**BA**	**PSO**	**BA**
1	589	543	913	840	1.082	963	1.635	1.452
2	890	818	1.200	1.104	1.474	1.297	1.933	1.701
3	1.081	995	1.416	1.302	1.861	1.638	2.515	2.213
4	-	-	-	-	2.311	2.011	2.662	2.467
5	-	-	-	-	2.662	2.316	3.487	3.034

**Table 18:** Performance comparison of the optimization- vs. classification-based approaches for analog circuit fault diagnosis.

#	Approach	Ref	A (%)	Better		Similar		Worst	
				**PSO**	**BA**	**PSO**	**BA**	**PSO**	**BA**
1	Radial Basis Function Network	[34]	65.77	**✓**	**✓**				
2	Bayesian Network	[34]	97.31				**✓**	**x**	
3	Support Vector Machine (SVM)	[34]	81.92	**✓**	**✓**				
4	Sparse Random Projections and K-Nearest Neighbor	[34]	100					**x**	**x**
5	Generalized Multiple Kernel Learning SVM	[35]	97.90				**✓**	**x**	
6	Generalized Multiple Kernel Learning SVM with PSO	[36]	98.30				**✓**	**x**	
7	Ensemble Empirical Mode Decomposition	[37]	98.64				**✓**	**x**	
8	Improved Ensemble Empirical Mode Decomposition with SVM	[38]	98.75				**✓**	**x**	
9	Proposed methodology based on PSO	-	93.93		**✓**	**✓**			
10	Proposed methodology based on BA	-	96.02			**✓**	**✓**		
